# Cytosine analogues as DNA methyltransferase substrates

**DOI:** 10.1093/nar/gkae568

**Published:** 2024-07-05

**Authors:** Marek Wojciechowski, Honorata Czapinska, Joanna Krwawicz, Dominik Rafalski, Matthias Bochtler

**Affiliations:** International Institute of Molecular and Cell Biology, Trojdena 4, 02-109 Warsaw, Poland; Plant Breeding and Acclimatization Institute - National Research Institute, 05-870 Radzikow, Poland; International Institute of Molecular and Cell Biology, Trojdena 4, 02-109 Warsaw, Poland; Institute of Biochemistry and Biophysics PAS, Pawinskiego 5a, 02-106 Warsaw, Poland; International Institute of Molecular and Cell Biology, Trojdena 4, 02-109 Warsaw, Poland; Institute of Biochemistry and Biophysics PAS, Pawinskiego 5a, 02-106 Warsaw, Poland; Department of Biochemistry, University of Oxford, Oxford, UK; International Institute of Molecular and Cell Biology, Trojdena 4, 02-109 Warsaw, Poland; Institute of Biochemistry and Biophysics PAS, Pawinskiego 5a, 02-106 Warsaw, Poland; International Institute of Molecular and Cell Biology, Trojdena 4, 02-109 Warsaw, Poland; Institute of Biochemistry and Biophysics PAS, Pawinskiego 5a, 02-106 Warsaw, Poland

## Abstract

DNA methyltransferases are drug targets for myelodysplastic syndrome (MDS), chronic myelomonocytic leukemia (CMML), acute myelogenous leukemia (AML) and possibly β-hemoglobinopathies. We characterize the interaction of nucleoside analogues in DNA with a prokaryotic CpG-specific DNA methyltransferase (M.MpeI) as a model for mammalian DNMT1 methyltransferases. We tested DNA containing 5-hydroxymethylcytosine (5hmC), 5-hydroxycytosine (5OHC), 5-methyl-2-pyrimidinone (in the ribosylated form known as 5-methylzebularine, 5mZ), 5,6-dihydro-5-azacytosine (dhaC), 5-fluorocytosine (5FC), 5-chlorocytosine (5ClC), 5-bromocytosine (5BrC) and 5-iodocytosine (5IC). Covalent complex formation was by far most efficient for 5FC. Non-covalent complexes were most abundant for dhaC and 5mZ. Surprisingly, we observed methylation of 5IC and 5BrC, and to a lesser extent 5ClC and 5FC, in the presence, but not the absence of small molecule thiol nucleophiles. For 5IC and 5BrC, we demonstrated by mass spectrometry that the reactions were due to methyltransferase driven dehalogenation, followed by methylation. Crystal structures of M.MpeI-DNA complexes capture the ‘in’ conformation of the active site loop for analogues with small or rotatable (5mZ) 5-substituents and its ‘out’ form for bulky 5-substituents. Since very similar ‘in’ and ‘out’ loop conformations were also observed for DNMT1, it is likely that our conclusions generalize to other DNA methyltransferases.

## Graphical Abstract

**Graphical Abstract:**
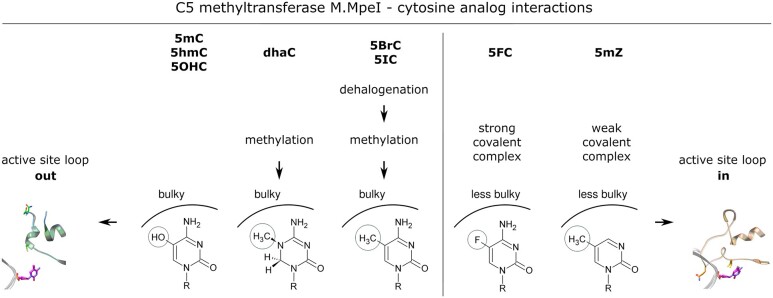

## Introduction

Nucleobase analogues of cytidine and 2′-deoxycytidine have attracted considerable interest as antineoplastic agents. After cell entry, the free nucleosides can be phosphorylated, and, if necessary, reduced by ribonucleotide reductase (1). Once incorporated into DNA, the analogues act as covalent or non-covalent methyltransferase traps (2). Their antineoplastic effects are attributed either to hypomethylation and re-expression of tumor suppressor genes (due to methyltransferase depletion and degradation) (3,4), or to preferential toxicity of DNA-protein crosslinks (DPCs) to rapidly proliferating malignant cells (4). 5-azacitidine (Vidaza, Azadine, Onureg) is used clinically for the treatment of some forms of myelodysplastic syndrome (MDS) (5), chronic myelomonocytic leukemia (CMML), (6) and acute myeloid leukemia (AML) (7,8). The 5-azacytidine derivative, 2ʹ-deoxy-5-azacytidine (Decitabine, Dacogen, Demylocan), is a more targeted DNA methyltransferase inhibitor than the parent compound, because it is incorporated only into DNA and not RNA (9). Decitabine is used clinically for similar indications as 5-azacytidine (10,11). 5,6-dihydro-5-azacytidine was originally synthesized as an inhibitor of nucleotide synthesis (12,13), but later found to be a substrate for ribonucleotide reductase (14). Once incorporated into DNA, it is a good inhibitor of C5 DNA methyltransferases, presumably acting as a transition state mimic (15). The compound has been tested in animal studies (16) and clinical trials (17), but is not routinely used. Zebularine (2-pyrimidinone riboside), a cytidine analogue lacking the exocyclic 4-amino group, serves as a potent demethylating agent in various cancer models (18–20). 2ʹ-Deoxy-5-fluorocytidine, a suicide mechanism inhibitor of DNA methyltransferases (21), also acts as an efficient hypomethylation agent (22), but suffers from toxicity associated with deamination (23). Recently, there has also been considerable interest in the development of non-nucleosidic inhibitors of DNA methyltransferases (24–27). Aside from a potential role as antineoplastic reagents, these inhibitors may also be used to reactivate fetal hemoglobin expression in β-hemoglobinopathies (25).

The primary target of hypomethylating cytidine analog drugs or prodrugs is the maintenance methyltransferase, DNMT1. Like other C5 methyltransferases, DNMT1 catalyzes methylation through the formation of a covalent protein-DNA intermediate resulting from the addition of an active site cysteine (of the so-called motif IV) to the cytosine C6 atom (with concomitant N3 protonation). Subsequently, the C5 atom of cytosine attacks the methyl carbon of the methyl donor S-adenosylmethionine (SAM) converting it to S-adenosylhomocysteine (SAH). β-Elimination of the active site cysteine residue from the C6 of cytosine, with concomitant loss of a proton from the C5 position, completes the reaction cycle (28,29) (Supplementary Figure S1). Kinetic isotope effect data suggest that the reaction proceeds sequentially, with methyl transfer as the rate limiting step (28). However, these studies are at odds with earlier work favoring a concerted mechanism and other rate limiting steps (30,31). Structurally, DNMT1 can adopt catalytically productive (32) and non-productive (26,33) conformations, depending on the arrangement of the active site loop. The non-productive DNMT1 conformation can be induced either by autoinhibition (33) or by a small-molecule interfering with the DNA binding (25,26).

M.MpeI is a prokaryotic CpG specific C5 cytosine DNA methyltransferase (34), and a good model for mammalian DNMT1, because of the same specificity. The DNA complexes of the two enzymes share key features. As for other DNA methyltransferases, amino acid intercalation (Met and Lys in DNMT1, Gln in M.MpeI) displaces the substrate cytosine from the stack of bases into the active site pocket. In contrast to most other DNA methyltransferases, M.MpeI and DNMT1 also unstack the CpG in the non-substrate strand, by additional amino acid intercalation (Trp and Lys in DNMT1, Phe in M.MpeI) (32,34). Thus, M.MpeI reproduces the features of the DNMT1 that are consistent with biochemical data, but not those that may be crystallization artifacts (35).

Here, we present a biochemical and crystallographic study of the interaction of M.MpeI with DNA containing various cytosine base analogs. In addition to cytosine and 5-methylcytosine (5mC) as controls, we tested 5-hydroxymethylcytosine (5hmC), 5-hydroxycytosine (5OHC), 5-methyl-2-pyrimidinone (in the ribosylated form called 5-methylzebularine, 5mZ), 5,6-dihydro-5-azacytosine (dhaC), 5-fluorocytosine (5FC), 5-chlorocytosine (5ClC), 5-bromocytosine (5BrC), and 5-iodocytosine (5IC). We analyzed covalent and non-covalent complex formation, and methyl transfer. We find that methylation of dhaC requires the methyltransferase, but is more efficient for a variant lacking the active site cysteine than for the wild-type enzyme. The 5-halocytosines, particularly 5BrC and 5IC, are methylated in a small molecule thiol nucleophile dependent manner, by a novel methyltransferase catalyzed dehalogenation reaction followed by methylation. Our structural data show that steric bulk in the 4 and 5 positions of the pyrimidine ring controls the conformation of the methyltransferase active site loop and the ease of enzyme-DNA complex formation.

## Materials and methods

### Site directed mutagenesis

In our studies, we used a previously described M.MpeI variant (34) that differs from the UNIPROT protein (accession Q8EVR5) by the Q68R, K71R, S295P point mutations (34). The Q68R and K71R changes relative to the UNIPROT sequence were naturally present in the genomic DNA template that we used to amplify the open reading frame. The S295P change was introduced deliberately, to suppress M.MpeI cleavage in the *Escherichia coli* expression host. Residue 295 is not in contact with DNA, and not part of the active site. It is therefore unlikely to have an effect on enzyme activity. For purification, an LEHHHHHH histidine tag was added to the C-terminus. Throughout the manuscript, we refer to this protein as the wild-type enzyme. The expression construct for the catalytic cysteine to alanine (C135A) mutation was prepared from the expression construct for the active protein by inside-out PCR using mutagenic DNA oligonucleotides. PCR product was digested with DpnI to degrade the template DNA, self-ligated and transformed into *E. coli* Top10 strain. The expression construct for the C135A variant of M.MpeI was sequenced to confirm the presence of the designed mutation and the absence of unintended ones.

### M.MpeI expression and purification

Recombinant M.MpeI and its catalytically inactive C135A variant were purified as previously reported (34). Briefly, *E. coli* strain ER2566 (New England Biolabs) was used for overnight expression of WT and variant M.MpeI at 16°C, induced by addition of IPTG to a final concentration of 0.1 mM. Cells were harvested by centrifugation and suspended in 20 mM Tris–HCl pH 7.5, 50 mM NaCl. Lysozyme, DNase I and PMSF were added, and the cells were incubated for 30 min at 4°C before disruption by sonication. The lysate was cleared by ultracentrifugation (190 000 × *g*, 20 min). The resulting supernatant was transferred to a beaker and placed on a magnetic stirrer at 4°C. An equal volume of 4 M (NH_4_)_2_SO_4_ was added dropwise, and the mixture was incubated for 1 h at 4°C. Precipitated proteins were removed by centrifugation (10 000 × *g*, 20 min) and the lysate was incubated overnight at 4°C to complete the salt-out process. The remaining precipitated proteins were removed by another centrifugation (10 000 × *g*, 20 min). The pH of the clear lysate containing M.MpeI was titrated to pH 7.5 by adding 1 M Tris–HCl pH 8.5 and then 1 M imidazole pH 7.5 to a final concentration of 20 mM. It was then loaded onto a Ni-NTA column (Qiagen). The column was extensively washed with wash buffer (20 mM Tris–HCl 7.5, 0.3 M NaCl, 40 mM imidazole, 0.01% 1-*O*-(*n*-dodecyl)-nonaethylene glycol). Purified M.MpeI was eluted with an elution buffer (20 mM Tris–HCl 7.5, 0.3 M NaCl, 0.2 M imidazole). Fractions containing M.MpeI were pooled, concentrated, and polished by a size exclusion chromatography run on a Superdex S75 column in 10 mM HEPES–NaOH (pH 8), 0.12 M NaCl, 5% glycerol (w/v). The active and inactive protein variants were purified with separate column sets and their activity or lack thereof was verified for each purification batch. Final protein purity was assessed by SDS-PAGE. For crystallization, the protein was purified in the presence of dithiothreitol (DTT). For biochemical studies, the protein was purified in the absence of DTT.

### Quality control of oligos with modified bases

Phosphoramidite building blocks for base analogues were purchased from Glen Research or Biosearch Technologies (Supplementary Table S1A). HPLC purified modified oligonucleotides were synthesized by either Purimex or FutureSynthesis (Supplementary Table S1B). The quality of modified oligonucleotides was analyzed by 20% PAGE in TAE buffer and detected with either Cy3 or GelRed (Supplementary Figure S2). The identity of modified bases was verified by degradation to single 2ʹ-deoxynucleoside monophosphates and dephosphorylation. The degradation reaction was performed in 10 mM Tris–HCl pH 8.8, 50 mM NaCl using 1 U of Viscolase nuclease (A&A Biotechnology), 1 U of phosphodiesterase and 1 U of bovine calf intestine phosphatase (CIAP) (APMB-RO Roche) for 15 hours at 37°C. Degraded samples were dehydrated in a SpeedVac and re-suspended in 25 μl ddH_2_O for LC–MS (20 μl injection loop). Samples were separated on an ACQUITY UPLC® Oligonucleotide BEH C18 column, (130 Å, 1.7 μm, 2.1 mm × 100 mm cartridge) in 0.1% formic acid solution in a dual linear acetonitrile gradient: 36 mins from 0 to 20%, followed by another 6 mins to reach 90%. Mass analysis was performed on a coupled SynaptG2 Waters Q-TOF mass spectrometer. The acquisition mass search range was 100–1000 Da in an ES^+^ ionization mode (Supplementary Figure S3).

### Oligonucleotide labelling

The non-substrate DNA strand was 5ʹ-end labelled by T4 polynucleotide kinase (NEB) using [gamma-^32^P] adenosine 5′-triphosphate (ATP) (Hartmann Analytic, Germany), purified on a BioSpin 30 gel-filtration column (Bio-Rad), and annealed with the substrate strand.

### M.MpeI-DNA covalent complex formation

Reactions were performed in 20 μl reaction buffer: 10 mM Tris–HCl, pH 7.9, 50 mM NaCl, 160 μM SAM or sinefungin (SFN). M.MpeI was used at a concentration of 3 μM, while the Cy3-labelled oligoduplex was used at a concentration of 3.75 μM (0.8 protein to DNA ratio). Dithiothreitol (DTT) was optionally used at a concentration of 10 mM. Reactions were incubated in a thermocycler at 37°C with the lid heated to 50°C. At the desired time point, the tubes were removed and the reactions were quenched by adding 5 μl of 5 × SDS-PAGE loading buffer without reducing agent (10% SDS, 50% glycerol, 0.25 M Tris–HCl, pH 6.8, bromophenol blue 0.1%). The samples were then resolved on a 10% SDS-PAGE gel until the dye front migrated out of the gel and visualized on an Azure 200 Gel Imager (Azure Biosystems). ThermoFisher PAGERuler Prestained Protein Ladder was used as a protein size marker (expected protein size is 47.31 kDa).

### M.MpeI–DNA complex formation under native and denaturing conditions

M.MpeI in increasing concentrations (0.5, 1, 2 and 4 μM) was incubated with modified oligoduplexes (1 μM) at 37 °C for 1 h in the buffer containing 10 mM Tris–HCl pH 7.9, 50 mM NaCl, 10 mM MgCl_2_, 1 mM DTT and 80 μM SAM. Samples were loaded onto 6% polyacrylamide TBE native gel either directly after incubation or after additional treatment with 1% SDS at 65 °C for 5 min. The gel-shift assay was performed for 1 h at 100 V, and then the gels were dried and used for autoradiography to monitor the presence of ^32^P labelled DNA.

### M.MpeI automethylation by SAM assayed on nitrocellulose filter

20 pmol M.MpeI (WT or C135A variant) was mixed with 80 μM ^3^H labelled S-adenosylmethionine (SAM, American Radiolabeled Chemicals, MO USA) in 20 mM HEPES–NaOH pH 7.5, 0.15 M NaCl, 0.2 mM EDTA and 2.5 mM MgCl_2_ buffer, which was previously degassed and argonized. The reaction was performed at 37°C for 15 h. The reaction mixtures were then transferred to a square nitrocellulose membrane (2 × 2 cm) (GE Healthcare, Amersham Protran, Premium 0.45 um NC), UV-crosslinked, washed four times for 5 min with 0.2 M phosphate buffer pH 6.5, once for 5 min in ddH_2_O, once for 5 min in 10% ethanol, and then dried. The presence of protein on the nitrocellulose membrane was independently confirmed by the α-His-tag Western blot. The dried membranes were placed in scintillation vials, each containing 2 ml of scintillation cocktail (Rotiszint R Eco Plus, LSC-Universal Cocktail, Roth). Incorporated ^3^H was counted using a Packard Bioscience liquid scintillation analyzer (TRI-CARB 2900 TR).

### Hybond filter assay for methyl transfer to dsDNA containing cytosine analogues

20 pmol of WT or C135A M.MpeI was mixed with 100 pmol of modified oligoduplexes analogous to SDS-PAGE analysis. The reaction was performed with 80 μM ^3^H labelled SAM in 20 mM HEPES–NaOH pH 7.5, 0.1 M NaCl, 0.2 mM EDTA and 2.5 mM MgCl_2_ buffer, optionally supplemented with 1 mM DTT. Methylation was performed in 30 μl volume for 1 and 3 h at 37 °C, and then stopped by adding 1% SDS. Next, 30 μl of reaction mixture was applied to a nylon membrane square (2 × 2 cm) (Hybond^TM^-N, GE Healthcare, RPN 203N), which was dried and washed 4 times for 5 min with 0.2 M phosphate buffer pH 6.5. The membrane was then washed with water for 5 mins, and finally with 70% ethanol. The dry membrane was transferred to scintillation vials and 2 ml of scintillation cocktail (Rotiszint^®^ Eco Plus, LSC-Universal Cocktail, Roth) was added. Incorporated ^3^H was counted using a Packard Bioscience Liquid Scintillation analyzer (TRI-CARB 2900 TR).

### RP-HPLC analysis of methylation products

0.1 nmol of WT or C135A M.MpeI was mixed with 1 nmol of oligoduplex (5′-TTCAG**5mC**GCATGTGG-3′, 5′-CCACATG**X**GCTGAA-3′, where **X** stands for C or 5BrC). The reaction was performed for 3 h at 37 °C in 20 mM HEPES–NaOH pH 7.5, 0.1 M NaCl, 0.2 mM EDTA and 2.5 mM MgCl_2_ buffer, optionally supplemented with 1 mM DTT. Reaction mixture was frozen and diluted 10 times before degradation into nucleosides. Digestion and dephosphorylation to single 2′-deoxynucleosides was performed in 10 mM NaCl, 75 mM Tris–HCl pH 8.8 by 1 U of Phosphodiesterase I from *Crotalus adamanteus* venom (Pharmacia Biotech) and 1 U of Polar Bacterial Alkaline Phosphatase (BAP) (EURx) for 16 h at 37 °C. The resulting mixtures were analyzed by HPLC. Reverse phase HPLC analysis (RP-HPLC) was performed using a Knauer dual pump system with a multi-channel UV spectrophotometer and a diode array technology detector managed by ClarityChrom controller V2.6.5.517. Waters Nova-Pak^®^ C18, 60 Å, 4 μm, 4.6 × 250 mm cartridge column at a flow rate of 0.7 ml/min with UV detection at the range from 215 to 450 nm was used for analytical separations. The linear gradient of 20 mM ammonium formate pH 6.5 × 20% acetonitrile over 45 min, followed by 15 min of 20% acetonitrile was applied.

### Demonstration of M.MpeI and small-molecule thiol-nucleophile driven dehalogenation

500 pmol of oligoduplexes (5′-TATAAA**X**GATATTT-3′, 5′-AAATAT**Y**GTTTATA-3′, where X stands for C, 5mC, 5BrC, 5ClC or 5IC and Y stands for either C or 5mC) was mixed with 50 pmol of WT or C135A M.MpeI. The reaction was performed in 20 mM of HEPES–NaOH pH 7.5, 0.15 M NaCl, 0.2 mM EDTA and 2.5 mM MgCl_2_ buffer. The reaction mix was optionally supplemented with reducing agent (3 mM DTT, 5 mM β-mercaptoethanol (βME) or 2 mM Tris(2-CarboxyEthyl)Phosphine (TCEP) and 160 μM SAM, SAH or SFN depending on the tested condition. Reactions were carried out at 37°C for 7 h (final time point done for all four halogenated bases), 0 and 7 h (end point assays for 5IC) and 0, 1, 3, 7 h (time courses for 5IC) and then terminated by heat inactivation at 95°C for 10 min. For end point assays, but not the time courses, DTT was re-added several times to the samples (with addition of equivalent volumes of buffer to samples without DTT), and buffers were flushed with argon, to counteract oxidative loss of DTT over time. The assays for final and end time points were performed in duplicate, the time course assays were done in triplicate. Degradation and dephosphorylation of DNA to 2′-deoxynucleosides, and analysis by LC–MS were carried out as described for the quality control of modified oligos. Chromatogram peaks were assigned based on their mass composition and comparison of the elution times with those of the authentic standards. Chromatograms were normalized against the peak area of G (a reference base not involved in any of the reactions of interest). For the presentation of mass filtered data, the signals for bases, SAM and SAH were separately normalized to peak heights of 100% in the controls.

### Demonstration of M.MpeI and small-molecule catalyzed C5-hydrogen exchange

The exchange assay was performed with 375 pmol of the same oligoduplexes as used for dehalogenation in the same buffers except for D_2_O instead of H_2_O. Reactions were run at 37°C for 7 h and then terminated by heat inactivation at 95°C for 10 min prior to degradation to nucleosides. Each reaction mixture was then dehydrated in a SpeedVac to stop the D_2_O exchange. Dry pellets were resuspended in degradation mix consisting of 10 mM Tris–HCl pH 8.8, 50 mM NaCl with 1 U Viscolase nuclease (A&A Biotechnology) and 1 U bovine calf intestine phosphatase (CIAP) (APMB-RO Roche) for 15 h at 37°C. Each sample was again dehydrated in the SpeedVac and resuspended in 25 μl ddH_2_O to fit in a 20 μl injection loop. Samples were separated for 30 min in a linear gradient of 0 to 100% acetonitrile in 0.1% formic acid on an ACQUITY UPLC® Oligonucleotide BEH C18 column (130 Å, 1.7 μm, 2.1 mm × 100 mm cartridge) coupled to a SynaptG2 Waters Q-TOF mass spectrometer with an acquisition mass search range of 100–1000 Da in ES+ ionization mode. Mass spectra were analyzed using MassLynx v4.1 software. The mass spectra (110–116 Da) corresponding to the top of the extracted peak for C (112 Da) were plotted and analyzed. The actual experiment was done in three repeats, the back exchange controls were performed once.

### M.MpeI crystallization and structure determination

Crystals of M.MpeI with DNA containing a selection of base analogues were grown as described previously (34). M.MpeI (5 mg/ml) in 10 mM HEPES–NaOH (pH 8.0), 0.12 M NaCl, 1 mM DTT, 5% glycerol (w/v), dsDNA (5′-CACATGXGCTGAA-3′ and 5′-GTTCAG5mCGCATGTG-3′; where X stands for the base analogue) in 10 mM Tris pH 8.0, and 32 mM SAM in 5 mM H_2_SO_4_, 10% ethanol were mixed 1:1:1. Crystals were obtained from the 1:1 mixture of the above with 10% PEG 3350, 0.15 M NaCl, and 50 mM sodium citrate (final pH 5.6) and cryoprotected with glycerol (25% v/v final concentration). The datasets of the highest resolution were collected at the P13 beamline of PETRA III (EMBL/DESY, Germany). The data were processed with XDS (36). The structures were solved by molecular replacement with the structure of M.MpeI in complex with the 5FC containing DNA (PDB ID: 4DKJ) (34). The models were rebuilt with ARP/wARP and refined with REFMAC (37) and COOT (38). Data collection statistics and final refinement parameters are shown in Table 1.

**Table 1.** Data collection and refinement statistics

	5mZ	dhaC	5OHC	5BrC
Wavelength (Å)	0.9184	1.2782	1.2782	0.9116
Unit cell dimensions				
*a*, *c* (Å)	84.26, 173.88	84.45, 172.56	84.52, 172.99	84.51, 173.12
Resolution (Å)	20–2.40	20–1.95	20–1.85	20–1.70
lowest shell	20–6.81	20–5.64	20–5.37	20–4.95
highest shell	2.54–2.40	2.07–1.95	1.96–1.85	1.80–1.70
Total reflections	217 518	161 542	307 494	612 927
Unique reflections	25 251	44 130	54 230	69 838
Completeness (%)*	99.6 (94.7, 99.0)	95.0 (81.2, 96.0)	99.5 (94.8, 99.0)	99.8 (97.9, 99.8)
Multiplicity*	8.6 (7.7, 8.8)	3.7 (3.7, 3.6)	5.7 (5.2, 5.7)	8.8 (7.9, 8.5)
I/σI*	16.9 (57.94, 2.05)	18.3 (55.7, 1.80)	21.8 (68.0, 1.97)	27.9 (90.9, 2.15)
*R*_sym_*	11.4 (2.8, 120.3)	3.7 (1.9, 70.1)	4.4 (2.1, 96.7)	3.9 (1., 91.9)
*R*_meas_*	12.1 (3.0, 127.7)	4.3 (2.2, 82.2)	4.8 (2.3, 106.7)	4.1 (2.1, 98.0)
CC_1/2_*	99.9 (100, 70.3)	99.9 (99.9, 68.5)	99.9 (99.9, 73.7)	100 (100, 82.2)
Solvent content^$^	56.4	56.3	56.4	56.5
B(iso) from Wilson (Å^2^)	53.3	48.6	43.5	38.4
*R*_work/_ *R*_free_	17.33/21.62	16.17/19.20	16.48/19.45	16.65/18.27
No. atoms^#^	4470	4610	4691	4716
Protein	3456	3501	3490	3452
DNA	569	570	570	570
Ligand/ion	33	73	79	99
Water	412	466	552	595
*B*-factors^#^				
Protein	51.5	47.4	42.7	36.8
DNA	56.0	53.1	49.0	43.4
Ligand/ion	55.2	60.9	56.4	50.9
Water	56.8	61.1	57.3	52.6
R.m.s. deviations				
Bond lengths (Å)	0.009	0.005	0.006	0.006
Bond angles (º)	1.01	1.09	1.16	1.14
Ramachandran				
allowed (%)	100.0	100.0	100.0	100
favored (%)	96.9	98.1	98.4	98.1
Molprobity clashscore	1.7	0.5	1.2	0.8
PDB code	8C56	8C57	8C58	8C59

*Lowest and highest resolution shell in brackets; ^$^protein and dsDNA included, SAM/SAH excluded; ^#^double conformations counted separately.

## Results

### Selection of dsDNA

**Figure 1.**
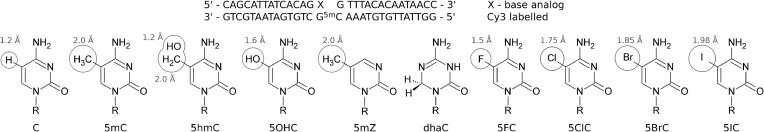
Oligonucleotides used for activity assays. The 30 base pair long oligoduplexes (top) contained the modified base (X) in one strand and 5-methylcytosine (5mC) in the other to prevent strand swap during the experiment. The strand containing 5mC was Cy3 labelled on the 5′ side. Theoretical van der Waals radii of 5-substituents based on the works of Richards (64) and Bondi (65) are indicated. dhaC has two key possible tautomers. Depending on the extent of calculations they either have similar free energies or the N3-H tautomer (shown) is favored by 2–3 kcal/mol (Supplementary Table S2).

In this work, we tested cytosine analogues as M.MpeI substrates (Figure 1). Chemically synthesized 2′-deoxyoligonucleotide duplexes were used. In the substrate strand, the modified cytosine was the only base in the CpG context. In the non-substrate strand, 5mC was used in the CpG dyad. 5mC in the non-substrate strand was chosen because hemimethylated DNA is the physiological substrate of DNA methyltransferases, and also because this choice prevented confounding effects of non-substrate strand methylation. The complete list of used oligonucleotides is provided in Supplementary Table S1.

### Formation of covalent M.MpeI–DNA complexes

**Figure 2.**
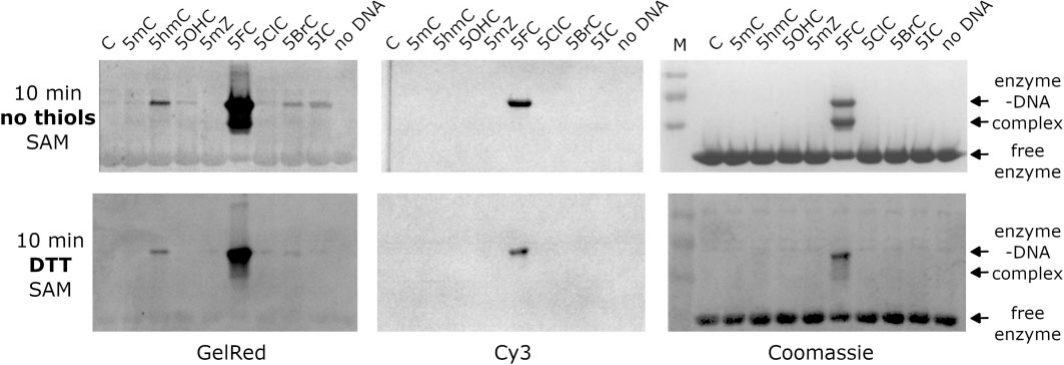
Covalent complex formation between M.MpeI and the modified oligoduplexes. The complex formation was monitored by PAGE. DNA was detected using GelRed straining (left) or direct Cy3 readout (middle), protein was detected by Coomassie staining (right).

C5 methylation of cytosine proceeds via a covalent enzyme-substrate intermediate involving the active site cysteine (Supplementary Figure S1) (28). With C as the substrate base, the covalent intermediate is very transient and difficult to detect. This need not be the case for C analogues, as demonstrated for 5FC, which acts as a suicide inhibitor and traps a covalent intermediate (21,39). To analyze the formation of covalent complexes, we incubated Cy3 labelled dsDNA with M.MpeI at 37°C, and subjected the reaction products to denaturing SDS-PAGE, in the absence or presence of thiol reducing agents (Figure 2, Supplementary Figures S4-S6). In our assay conditions, protein, as well as protein-DNA complexes migrated into the gel at different rates, while faster migrating DNA eluted from the gel. Protein was visualized by Coomassie staining and DNA by GelRed staining or fluorescence of the same gel. Already at the earliest time point tested, 10 min after mixing protein and DNA, we observed a prominent upshifted band for the complex of M.MpeI with 5FC containing DNA, as expected (39) (Figure 2). In the absence of DTT, much weaker bands of covalent complex with the oligoduplexes containing 5hmC and 5OHC, and also 5BrC and 5IC, were observed. At the 10 min time point, these weaker bands were only detectable by the very sensitive GelRed staining, but not Cy3 fluorescence alone (Figure 2 top). At later time points, sufficient amounts of complexes accumulated to be detected by Cy3 fluorescence or Coomassie staining not only for 5FC, but also 5OHC and 5IC (Supplementary Figure S4). 5mZ complex consistently migrated differently from other complexes for unclear reasons. The presence of DTT reduced the amounts of covalent complexes, presumably due to disulfide exchange with the active site cysteine (Figure 2 bottom, Supplementary Figure S5). While a strong signal was still observed for the covalent complex with 5FC, the signal for 5BrC and 5IC DNA was largely lost. Interestingly, the covalent complexes with 5hmC and 5OHC DNA appeared to be less sensitive to DTT than the complexes with 5BrC and 5IC DNA. At least in the presence of DTT, the formation of covalent complexes was dependent on the presence of SAM, as reported earlier (40) and not observed when SAM was replaced by sinefungin (a naturally occurring SAM analogue and methyltransferase inhibitor (41)) (Supplementary Figure S6).

### Formation of non-covalent M.MpeI–DNA complexes

We incubated M.MpeI with DNA in the presence of SAM and DTT and analyzed the complex formation by non-denaturing PAGE, followed by the ^32^P radiolabelled DNA detection (Supplementary Figure S7). Prior to loading of M.MpeI mixtures on the gel, we applied an optional heating step. Without the heating step, non-denaturing PAGE detects both covalent and non-covalent complexes. The heating step is expected to denature M.MpeI, so that only covalent complexes should be observed, as in the earlier analysis, when protein was denatured by the SDS in the gel electrophoresis buffer. When heat denaturation was applied, major complex formation was only seen for 5FC DNA, in agreement with the results obtained by denaturing PAGE. By contrast, when the heating step was omitted, strong upshifted bands were observed for many of the analogues, including dhaC, 5mZ, 5FC and 5OHC. While the 5FC complex is likely predominantly covalent, the other complexes are expected to be predominantly (5OHC) or entirely (dhaC) non-covalent, since the covalent complexes are very scarce.

### Methyl transfer to dsDNA

**Figure 3.**
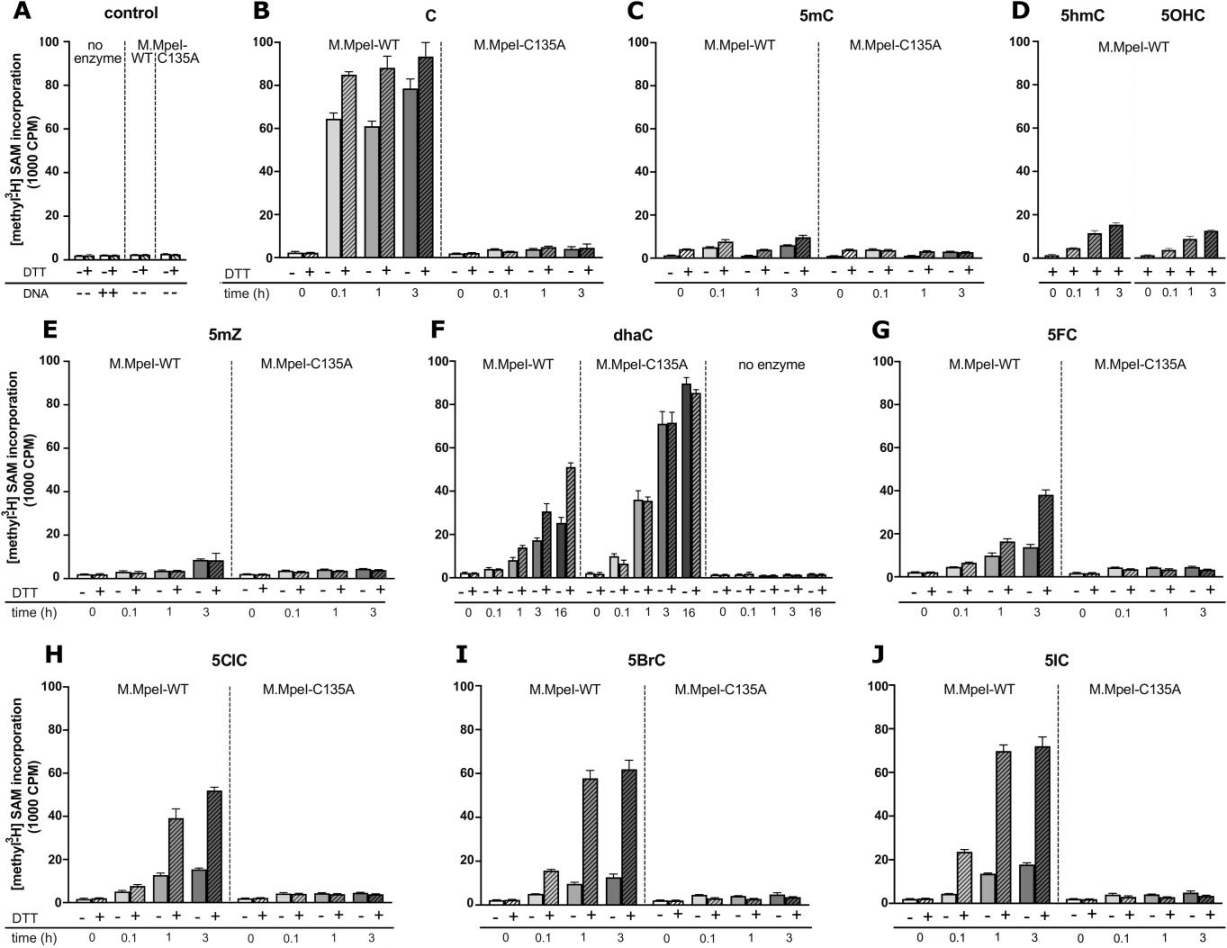
Radioactive assay of M.MpeI catalyzed methylation of modified DNA. *S*-Adenosylmethionine (SAM) methyl donor was ^3^H labelled. SAM and DNA were separated by filter binding or ethanol precipitation. The transfer of the radioactive CH_3_ group was monitored with a scintillation counter. Multiple-turnover conditions (five-fold stoichiometric excess of dsDNA over M.MpeI) were used. The experiment was repeated three times, with two technical replicas each. The error bars corresponding to SD were calculated with Prism GraphPad Software.

M.MpeI catalyzed methyl transfer from ^3^H labelled SAM to dsDNA containing cytosine analogues was measured using a substrate that contained only a single cytosine analogue in the CpG context in the substrate strand, and a 5mC base in the non-substrate strand. Methyl transfer was monitored by a filter binding assay that measured the radioactivity of protein-DNA complexes or DNA retained on a Hybond filter after extensive washing to remove SAM. Control experiments confirmed that the enzyme remained fully active for the duration of the assay and beyond (Supplementary Figure S8), and that automethylation of M.MpeI was minimal (Supplementary Figure S9). In the absence of DNA and protein, we observed only background levels of radioactivity (Figure 3A). They were likely due to unspecific methylation of the Hybond filter by SAM (or SAM retention on the filter), and not to methyltransferase automethylation, because protein addition had little effect (Figure 3A). Fortunately, the background in the methyltransferase assay was small not only compared to the signal for cytosine methylation, but also for methyl transfer to most modified bases (Figure 3B–J). Methyl transfer to C was slightly more efficient in the presence of DTT than in its absence, presumably due to a greater fraction of enzyme with reduced and therefore nucleophilic, active site cysteine. As expected, the C135A M.MpeI variant lacking the catalytic cysteine, transferred radiolabelled methyl only at near background levels (Figure 3B).

### Small, active site cysteine dependent methyl transfer to 5mC, 5hmC, 5OHC and 5mZ

Time dependent methyl transfer to DNA containing 5mC, 5hmC, 5OHC and 5mZ (Figure 3C–E) occurred at much lower levels than for C in the CpG context (Figure 3B). The transfer was clearly above background, depended on an intact methyltransferase active site, and increased with time. Since the extent of the methyl transfer differed, it was implausible to result purely from the methylation in contexts other than CpG. 5mC demethylation, described previously for mammalian methyltransferases (46), followed by enzyme dependent remethylation, could be the alternative. The most pronounced methyl transfer to 5hmC may be explained by a dehydroxymethylation reaction, reported for other DNA methyltransferases in the absence of SAM (42), followed by re-methylation with radioactive SAM as the donor.

Mechanistically, such exchange is reminiscent of thiol catalyzed hydrogen isotope exchange at the C5 position of pyrimidines (43–45). To test the plausibility of this explanation, we monitored the stability of the cytosine C5 hydrogen in the presence and absence of methyltransferase. 3 mM DTT alone was insufficient to catalyze detectable isotope exchange. However, wild-type M.MpeI, but not its inactive C135A variant, catalyzed a robust mass increase of 1 Da for cytosine (from the MH+ mass of 112 to 113), consistent with exchange of the C5 hydrogen for deuterium. The exchange occurred in the presence and absence of DTT. As the +1 upshift was much more prominent than the +2 or +3 upshifts, the data also indicate that the exchange was more rapid at the C5 carbon than at the exocyclic N4 amino group of cytosine (Supplementary Figure S10). We conclude that the methyl transfer to the 5hmC substrate presumably followed the methyltransferase catalyzed loss of the hydroxymethyl group as formaldehyde.

### Robust, active site cysteine independent methyl transfer to dhaC

Methyl transfer to dhaC occurred on a scale comparable to C. Among all the analogues, dhaC was the only one that was better methylated by the C135A variant of M.MpeI than by the wild-type enzyme itself. The robust methylation catalyzed by the inactive enzyme was DTT independent. In the absence of enzyme, only background levels of methyl transfer were observed (Figure 3F). All these observations can be explained in terms of the unusual properties of dhaC in the series. According to its chemical structure, dhaC is the only non-aromatic compound, as evidenced also by the clear deviation from planarity of the six-membered ring in the crystal structure (see below). A recent study suggests a pKa value ∼7 for dhaC (47), indicating that about half of the dhaC bases are non-protonated at neutral pH. The neutral form could exist in two tautomeric forms, with the proton either on the N3 (as in Figure 1), or the N5 nitrogen. Roothaan-Hartree-Fock (RHF) and Density Functional Theory (DFT) calculations in water indicate an equilibrium of both forms, with a preference for the N3-H tautomer at higher levels of theory (Supplementary Table S2). This leaves the N5 with an intrinsically nucleophilic lone pair of electrons for attack on the methyl group of SAM, independent of the M.MpeI active site cysteine.

The lower activity of the wild-type enzyme compared to the C135A variant can be attributed to steric hindrance. An approach of the active site cysteine to the C6 is not favorable (because the C6 is not nucleophilic) and sterically hindered by a conflict between the cysteine thiolate and one of the C6 hydrogen atoms. The steric conflict is avoided when the active site cysteine is replaced by a less bulky alanine residue, presumably allowing for proper positioning of dhaC relative to SAM. While the active site cysteine is not required for the methyl transfer reaction, the enzyme is. In the absence of M.MpeI, only background levels of methyl transfer were observed, suggesting that the enzyme (or its inactive variant) may be required to bring dhaC and SAM into spatial proximity (Figure 3F). We note that our observation that dhaC acts as a slow methyltransferase substrate is fully consistent with earlier descriptions of dhaC as a methyltransferase inhibitor (15).

### Substantial, active site cysteine and DTT dependent methyl transfer to 5ClC, 5BrC and 5IC

Robust transfer of methyl was also observed for the 5-halogenated cytosine analogues in the presence of DTT, and to a much lesser extent in its absence. The methyl transfer was dependent on both time and presence of the active site cytosine of M.MpeI. The efficiency of the transfer reaction increased in the order 5FC, 5ClC, 5BrC, 5IC (Figure 3G–J). As we used a five-fold excess of DNA over protein in our assays, at most 20% of the transferred radioactivity (relative to the cytosine control) could be explained by a covalent suicide complex. For 5ClC, 5BrC and 5IC, the extent of methyl transfer was clearly greater than 20%, indicating that some turnover had taken place. The effect was least pronounced for 5FC. The additional control experiments with fixed amounts of 5FC DNA and varying amounts of M.MpeI did not show clear excess over stoichiometry either (Supplementary Figure S11). Thus, we confidently infer excess methylation above the level that is expected for the suicide complexes only for 5ClC, 5BrC and 5IC.

**Figure 4.**
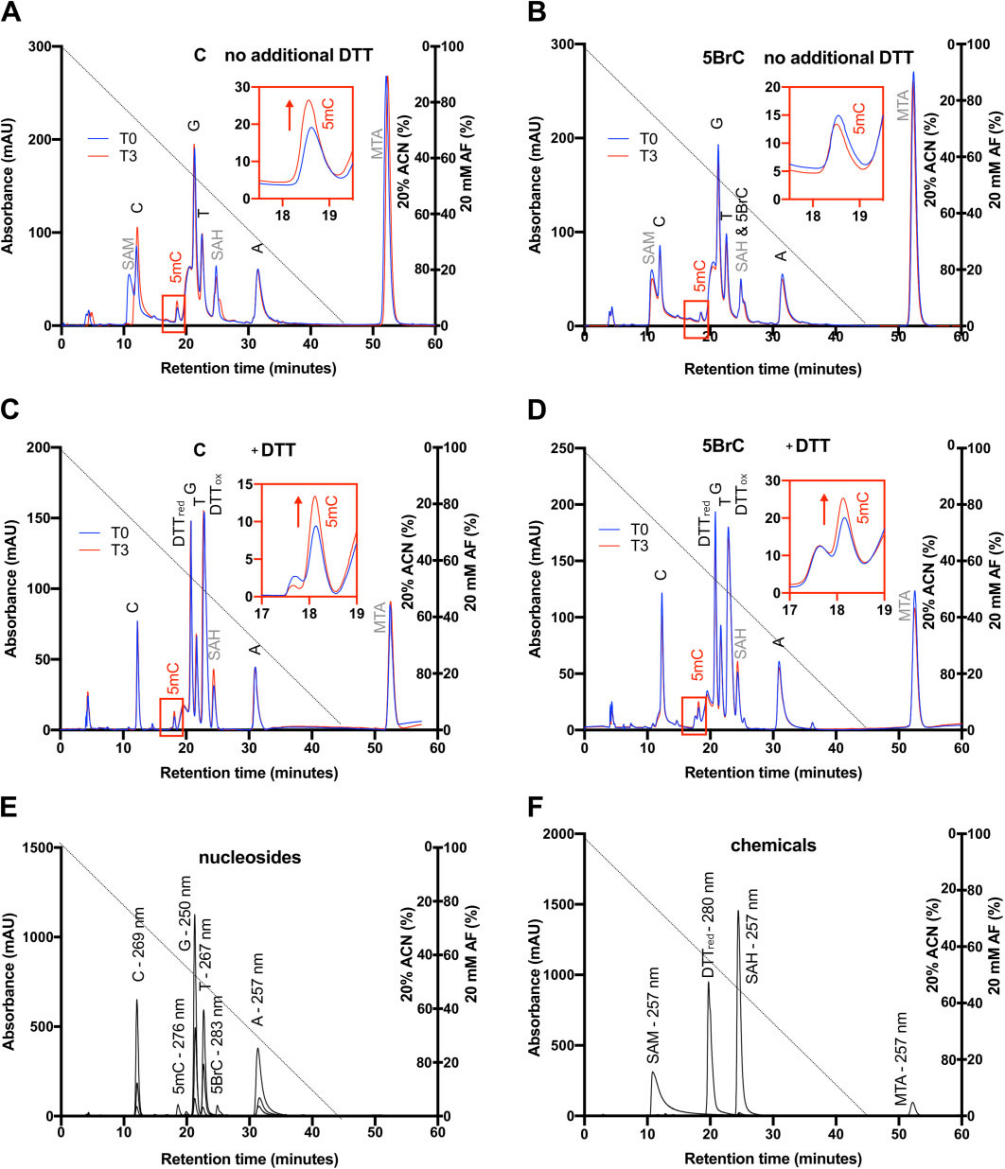
HPLC assay of M.MpeI catalyzed methylation of C and 5BrC containing DNA. M.MpeI treated dsDNA was digested and dephosphorylated to single 2′-deoxynucleosides. Authentic standards were used for peak assignment. A small amount of 5-methyl-2′-deoxycytidine was observed in all cases due to the presence of 5mC in the complementary DNA strand. ACN - acetonitrile, AF - ammonium formate.

Based on the reports of thiol nucleophile driven dehalogenation in the chemical literature (48–50), we suspected that the methyl transfer beyond the expected level for the suicide complex may be attributed to dehalogenation followed by methylation. To check the hypothesis, the 5BrC containing oligoduplex was treated with M.MpeI, SAM and optionally DTT, digested to 2′-deoxynucleoside monophosphates, dephosphorylated and analyzed by HPLC. Peaks were assigned based on a comparison to authentic standards, taking both retention times and UV-absorption profiles into account. In all elution profiles, we observed a small peak for 5-methyl-2′-deoxycytidine, due to the presence of 5mC in the bottom strand. For the 5BrC containing DNA, the height of this peak remained unchanged when the substrate was incubated with M.MpeI and SAM in the absence of DTT, and approximately doubled in the presence of DTT, as would be expected for 5BrC to C conversion, followed by methylation (Figure 4).

### M.MpeI and thiol-nucleophile dependent dehalogenation of 5BrC and 5IC

**Figure 5.**
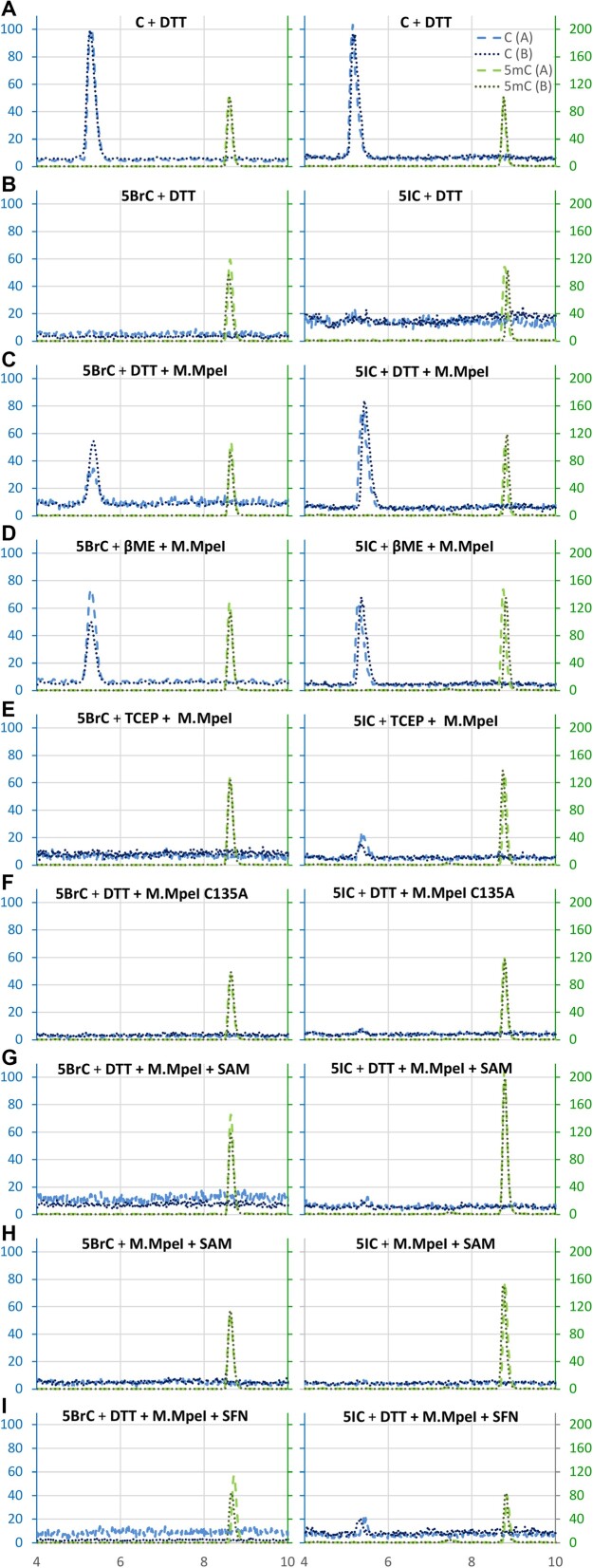
M.MpeI and small molecule thiol nucleophile driven dehalogenation of 5BrC and 5IC. Dehalogenation reactions or controls were subjected to degradation to single nucleotides, followed by dephosphorylation and analyzed by LC–MS. The indicated LC traces for C and 5mC are filtered for the expected masses. All reactions were scaled to equalize the signal from G (a base not involved in any reaction). Ordinate values are chosen to have a peak height of 100% for the C or 5mC control, respectively. The x-axes correspond to the elution time (min). More comprehensive MS results are presented in Supplementary Figures S12 and S13.

The HPLC demonstration that 5BrC was converted to 5mC in the presence of M.MpeI, SAM and DTT was consistent with our hypothesis of M.MpeI and DTT driven dehalogenation, but did not directly test the proposed reaction pathway with a cytosine intermediate. To test the hypothesis more rigorously, we designed second-generation, shorter oligoduplexes, which did not contain any C in either stand, and had only a single 5mC in CpG dyad in the non-substrate strand. Oligoduplexes were incubated with M.MpeI or its inactive variant, DTT or β-mercaptoethanol (βME) as thiol reducing agents, or Tris(2-CarboxyEthyl)Phosphine (TCEP) as their non-thiol alternative, and optionally SAM. Reaction products were again digested, dephosphorylated and analyzed by LC–MS. In the chromatography traces, we saw signals for single 2′-deoxynucleosides, as well as their dimers, and signals from the free bases, as a result of glycosidic bond cleavage under mass spectrometry conditions. Within an experimental series, normalization was done with respect to the total amount of G, followed by a correction for the unequal detection efficiencies of different bases. As signals were relatively weak, separate filtering was applied for the expected masses of C, 5mC and the halogenated bases and the peaks were again normalized with the signal estimated from the control experiments (Figure 5 and Supplementary Figure S12).

In all cases, a distinct peak for 5mC was observed, that was due to the presence of 5mC in the non-substrate strand (Figure 5, green). The very similar height of the 5mC peaks for reaction mixes not including SAM (in which 5mC could not be newly formed) served as an internal control for the normalization. In a control reaction with C in the substrate strand, a clear peak for C was observed (Figure 5A, blue traces). No such peak was seen when C was replaced with 5BrC or 5IC, indicating that pure chemical dehalogenation by thiol nucleophiles as reported in the chemical literature (48–50) was insignificant in our assays (Figure 5B). However, when active M.MpeI was additionally present (without SAM), a clear peak for C was observed (Figure 5C). A very similar peak was present when DTT was replaced with βME (Figure 5D). Both DTT and βME can be used as reducing agents or thiol nucleophiles. To test whether the presence of small molecule thiol nucleophile was required or whether a reducing agent was sufficient, we replaced DTT with TCEP. In this reaction, no peak for C was observed for 5BrC and only a small peak for 5IC (Figure 5E), confirming that thiol nucleophiles promoted the reaction. The reaction was dependent on the M.MpeI active site, since the peak for C was lost when the M.MpeI C135A variant was used (Figure 5F).

In a reaction with M.MpeI, DTT and SAM, methylation rapidly converted any C from 5BrC or 5IC dehalogenation to 5mC. Our measurements were not accurate enough to see any potential acceleration of the dehalogenation reaction in the presence of SAM according to Le Chatelier's principle (51) or by other effects of SAM on the dehalogenation reaction. In the case of 5IC, the dehalogenation-methylation reaction was sufficiently efficient that the 5mC peak was noticeably increased, indicating that a considerable fraction of the 5IC was converted to 5mC (Figure 5G). This increase was much less pronounced, when DTT was omitted (Figure 5H). Interestingly, the addition of the methyltransferase inhibitor sinefungin suppressed the C peak that would otherwise be expected in the presence of wild-type M.MpeI (Figure 5I). For 5ClC containing DNA, the C intermediate was not detected in any of the reaction conditions. As 5FC and 5ClC were methylated much less efficiently than 5BrC and 5IC in the presence of DTT, it is possible that the lack of a signal was simply a sensitivity issue. In the case of 5FC, methyl transfer could occur to the trapped covalent 5FC-enzyme intermediate, bypassing the need for dehalogenation.

We next validated all observations with the time-controlled experiments performed on 5IC-containing DNA, where dehalogenation was most pronounced. MS analysis covering the start and end point of the reaction confirmed all previously observed effects (Supplementary Figure S13A). Time course experiments supported the key observation that in the absence of SAM, SAH, or sinefungin, there was a time-dependent decrease of 5IC and increase of C. In the presence of SAM, we observed a time-dependent decrease of 5IC and of SAM, and increase of SAH and 5mC (Supplementary Figure S13B). Since the suppression of dehalogenation by sinefungin was surprising, we additionally tested the reaction in the presence of SAH instead of SAM. As before, we observed hardly any newly generated C, although the amount of 5IC decreased significantly. Suppression of 5IC dehalogenation by sinefungin or SAH was reminiscent of the suppression of covalent M.MpeI complex formation with 5FC containing DNA, but whether the two effects were mechanistically related remains unclear (Supplementary Figure S13A).

### Co-crystal structures of M.MpeI with dsDNA containing cytosine analogues

**Figure 6.**
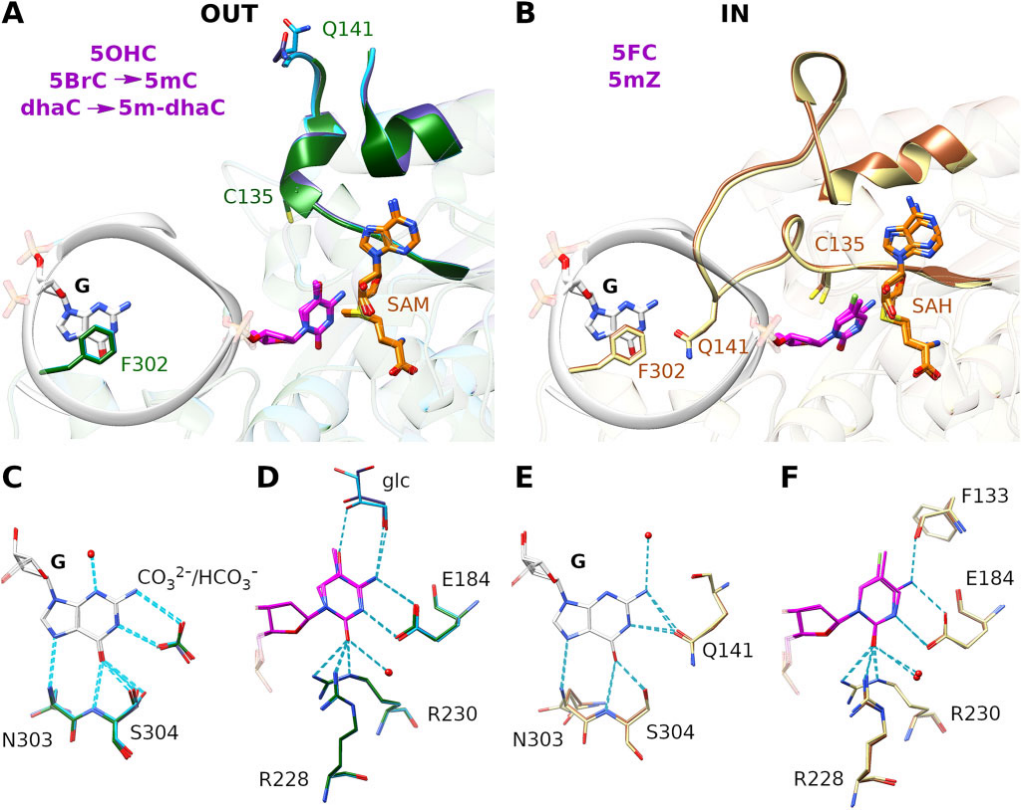
‘In’ and ‘out’ conformations of the active site loop in the M.MpeI-analogue complexes. The overlay of the analogues enforcing the loop ‘out’: 5OHC, 5BrC converted to 5mC, and dhaC converted to 5m-dhaC **(A, C, D)**; and loop ‘in’ conformation: 5mZ and 5FC (34) **(B, E, F)**. Overall view of the DNA binding region (A, B), zoom in on the estranged base (C, E) and the flipped-out substrate/product base (D, F). The separate views of the complexes together with the corresponding composite omit maps are shown in Supplementary Figure S14.

M.MpeI was co-crystallized with dsDNA fragments in reported conditions (34). The duplexes were shorter than for biochemical experiments and contained the cytosine analogue (5OHC, 5mZ, 5BrC or dhaC) in the ‘substrate’ strand and a 5mC base in the non-substrate strand. All co-crystallization experiments were set up in the presence of SAM, but if a reaction took place, the co-substrate could be converted to SAH. Note that SAM is unstable at neutral pH and can decompose by an intramolecular reaction to 5′-methylthioadenosine (MTA) and homoserine lactone (52). M.MpeI DNA crystals diffracted to a resolution better than 2 Å, except for 5mZ complex, which diffracted only to 2.45 Å. The density for SAM or SAH was typically better resolved in the nucleoside region, and the protein anchored methionine ‘main chain’, but poorly defined for its side chain, probably reflecting the presence of a mixture of SAM, SAH and MTA in the crystals. Key features of the M.MpeI structure in complex with 5FC containing DNA (34) are conserved in the co-crystals with other analogues. In the substrate strand, the cytosine analogue is extruded from the stack of DNA bases. It is accommodated in a pocket of the enzyme, so that its Watson-Crick edge engages in hydrogen bonding with the carboxylate group of the active site Glu184 residue. In the non-substrate strand, the CpG dinucleotide step is unstacked by intercalation of Phe302 (Figure 6 and Supplementary Figure S14).

### Fate of the DNA base analogues in the co-crystal structures

The 5OHC and 5mZ bases appear in the complex structures as in the chemically prepared oligoduplexes. In the co-crystals grown with 5BrC containing DNA, the electron density indicates a planar ‘substrate’ base, suggesting that the aromaticity of the analogue was preserved. However, the density near the C5 position is consistent with a methyl group (15 electrons), but not a bromine substituent (35 electrons). The absence of an anomalous signal supports the conclusion that in the co-crystal the C5 substituent is a methyl group, rather than a bromine atom. Similar analysis indicated that the cytosine analogue in the non-substrate strand is also 5mC and not 5BrC, ruling out the DNA bound in the ‘reverse’ mode, with 5BrC in the non-substrate strand. Together, the crystallographic findings suggest that the 5-bromo substituent in the crystals had been replaced by a methyl group (Supplementary Figures S15 and S16). Since M.MpeI was polished for crystallization by a gel filtration step in a DTT containing buffer, this finding is consistent with the biochemical result that the enzyme transfers methyl in the presence of DTT (Figure 3I). In the co-crystals of M.MpeI with dsDNA containing dhaC, the electron density indicates that methylation of the N5 imino group had occurred to a significant extent during crystal growth (Supplementary Figure S17). This was consistent with the observation that dhaC was methylated not only by the C135A M.MpeI variant, but also by the wild-type enzyme (Figure 3F).

### ‘In’ and ‘out’ conformations of the active site loop

The M.MpeI DNA co-crystal structures are similar overall, except for drastic differences in the region surrounding the active site cysteine (residues 132–157). For this region of the protein, two very different conformations were observed, an ‘in’ conformation placing the active site cysteine near the C6 atom of the substrate cytosine analogue, and an ‘out’ conformation, placing the cysteine far away. The ‘in’ conformation was observed for M.MpeI in complex with DNA containing 5mZ and 5FC (reported previously (34)). All other structures exhibited the ‘out’ conformation (Figure 6).

The M.MpeI ‘out’ conformation has a rather accessible active site. It is therefore likely to represent the enzyme at the beginning of the reaction before the active site loop closes in for catalysis and covers the active site. In the crystal, it is either the product of a failure of the active site loop to shift to the ‘in’ conformation, or the result of a frustrated attempt to eject product-like bases from the active site pocket. Since this is not possible in the crystal lattice, the active site loop yields instead. When the loop in the ‘in’ conformation is modelled in the structures captured in the ‘out’ conformation, substantial steric clashes with the Cβ and Sγ atoms of the catalytic cysteine are observed, suggesting that they help promote the ejection of the product base (Supplementary Figure S18). In this light, and in agreement with previous proposals for a steric conflict in case of other methyltransferases (53–55), it is readily understood that 5mC (from the reaction with 5BrC), 5OHC and (methylated) dhaC induce the ‘out’ conformation. In the latter case, the ‘in’ conformation is also excluded by a steric clash of the attacking active site cysteine (Cys135) with one of the C6 hydrogen atoms of the analogue.

The M.MpeI ‘in’ conformation has an inaccessible active site and probably traps the catalytically productive state. It is observed for 5FC (34), and surprisingly also for 5mZ containing DNA in complex with M.MpeI. In this form, Cys135 is poised for the nucleophilic attack on the C6 atom of the substrate base. The distances between the cysteine Sγ and the C6 carbon of the analogue are 2.9 Å for 5FC and 2.6 Å for 5mZ. These values are intermediate between a covalent bond (1.8 Å) and the sum of the van der Waals radii of carbon and sulfur (3.5 Å). Thus, the crystals may contain a reaction intermediate or a mixture of complexes with different degrees of covalent bond formation. The ‘in’ conformation is observed in the 5mZ complex despite the presence of the 5-methyl group. Steric conflict is in this case avoided due to the greater rotational freedom of 5mZ compared to the other bases. For the natural base cytosine and the other analogues, the N4 amino group hydrogen bonds with the side chain of Glu184, and a main-chain carbonyl oxygen of Phe133. The interactions fix the plane of the base. 5mZ, which lacks this amino group, is freer to rotate and therefore can avoid the steric clash between the methyl group and the active site loop in the ‘in’ conformation.

The choice between the ‘in’ and ‘out’ conformations affects the interactions with the estranged guanine base in the DNA (originally paired with the substrate cytosine or one of its analogues). For the ‘in’ conformation, Gln141 accepts a hydrogen bond from the N2 of the Watson-Crick edge of the guanine. In the amino acid sequence, Gln141 is close to Cys135 and belongs to the mobile loop. Thus, in the ‘out’ conformation, this residue is placed away from the estranged guanine. Instead, we see electron density, not due to a protein side chain, in the vicinity of the Watson–Crick edge of the estranged base. Guided by its shape, we have tentatively interpreted this electron density as a bicarbonate ion. Interestingly, the fate of the loop is also correlated with the choice of the cofactor. SAH or a mixture of SAM and SAH is present in the complexes with the loop in the ‘in’ conformation. In contrast, the loop ‘out’ conformation allows for the replacement of the cofactor with SAM for which the enzyme has much higher affinity.

## Discussion

### Covalent methyltransferase-DNA complexes

The mechanism of action of 5FC as a suicide inhibitor of methylation and the formation of a covalent intermediate between the oligoduplexes containing this base and DNA methyltransferases are well known (21). A strong covalent complex of M.MpeI with 5FC DNA was observed in the presence and absence of DTT (Figure 2). For other cytosine base analogues, more covalent protein-DNA complexes were formed in the absence of DTT than in its presence (Supplementary Figures S4-S5). The loss of the covalent complexes upon DTT addition was essentially complete for 5ClC, 5BrC and 5IC, whereas the complexes with 5hmC and 5OHC were more resistant to the presence of the thiol reducing agent (Supplementary Figure S5). We suggest that the DTT effect is caused by a nucleophilic substitution reaction at the C6 atom of the cytosine analogue, with the thiol acting as a nucleophile. This interpretation is consistent with the 5ClC, 5BrC and 5IC complexes being more DTT labile than those of other compounds. Electron abstraction by the halogen atoms should keep the C6 carbon of 5ClC, 5BrC and 5IC electrophilic for DTT attack, presumably facilitating the resolution of complexes in the presence of the thiol reducing agent. Several previous reports have shown that zebularine forms a covalent complex with DNA methyltransferases (53,56,57). We expected this observation to generalize to 5mZ. And indeed, we could observe a clear signal for a DTT labile complex after overnight incubation of M.MpeI with 5mZ DNA in the presence of SAM. Surprisingly, this complex migrated differently (faster) than the other covalent complexes in SDS PAGE gel electrophoresis. We currently do not have an explanation for its anomalous migration.

### Non-covalent methyltransferase–DNA complexes

Non-covalent complexes are detected for all cytosine analogues by highly sensitive autoradiography, presumably because of general affinity of M.MpeI for target DNA. Prominent non-covalent complexes of M.MpeI were observed for 5mZ, dhaC and 5OHC (Supplementary Figure S7). Zebularine was previously shown to form covalent complexes with DNA methyltransferases (53,56,57), but also to have a strong tendency for non-covalent ones (53). Apparently, both properties are shared by 5mZ. In our hands, the covalent complex between M.MpeI and 5mZ is only a minority species. Most of the M.MpeI-5mZ complex in the absence of DTT, and all or almost all of it in the presence of DTT is non-covalent (Supplementary Figure S7).

From a structural point of view, one might expect more non-covalent complex formation for analogues that adopt the ‘in’ conformation of the active site loop (associated with the bound substrate) than for those that enforce its ‘out’ form (associated with the product ejection). 5mZ allows the ‘in’ loop conformation despite the presence of a 5-methyl group, because the absence of the amino group reduces the steric bulk and increases the mobility of the nucleobase in the pocket of the enzyme. dhaC also has little steric bulk in the 5-position of the pyrimidine. In the crystal structures, dhaC enforces the loop ‘out’ conformation, because methylation takes place and adds steric volume to the N5. The dhaC methylation reaction occurs on a timescale of hours (Figure 3F). The slow process is sufficient to drive methylation to completion or near completion during the long period of crystal growth, but it is insufficient to methylate the base during the time allotted for complex formation in the biochemical experiment (Supplementary Figure S7). Therefore, is likely that the dhaC complex is also an ‘in’ complex, before becoming an ‘out’ complex at later time points. If so, then there is a correlation between the tendency to form the ‘in’ conformation and the ability to form strong non-covalent (5mZ, dhaC) or covalent (5FC) complexes.

### Reaction mechanism for small molecule thiol nucleophile and M.MpeI dependent methylation of 5-halogenated cytosines

We were initially puzzled by M.MpeI catalyzed, robust methylation of 5BrC and 5IC (and to a lesser extent 5ClC) in the presence of DTT or βME, beyond the level that could be expected for the suicide complex (Figure 3H–J). The solution to the puzzle was found in the chemical literature, which contained evidence for small molecule thiol nucleophile dependent dehalogenation, including the cysteine catalyzed process (48–50). In our reaction conditions, with low concentrations of DNA, we did not observe purely chemical dehalogenation with small molecule nucleophiles DTT and βME (Figure 5B). However, we detected robust dehalogenation in the presence of M.MpeI (Figure 5C-D), but not its C135A variant (Figure 5F), strongly suggesting that the active site cysteine played the role of the small molecule thiol nucleophile from the chemical literature. Compared to small molecule thiols, the cysteine of the methyltransferase has the advantage of a high effective concentration in proximity to 5BrC or 5IC, as a consequence of protein-DNA binding. In the previously described dehalogenation reactions, the small molecule thiols react twice. First, they carry out the initial nucleophilic attack, which appears to be performed by the M.MpeI active site cysteine in our assay. Then, they react a second time to promote the resolution of the complex formed by the initial nucleophilic attack (48–50). In this second reaction, a cysteine of another M.MpeI molecule cannot substitute for the small molecule thiol nucleophile due to steric hindrance. Therefore, we suggest that the small molecule nucleophile is required for the resolution of the covalent enzyme-DNA complex. This resolution can take one of several mechanistic paths.

Initial formation of the covalent complex could be followed by HBr or HI elimination, resolution of the covalent complex by the attack of a thiol nucleophile on the cysteine sulfur and disulfide exchange (Supplementary Figure S19A). Alternatively, resolution of the covalent intermediate may occur by mechanisms previously proposed for dehalogenation by small molecule thiol nucleophiles alone (58). In one scenario, a second thiol could attack the halogen atom to form sulfenyl halide, thereby resolving the covalent complex (Supplementary Figure S19B, top). In another scenario, initial addition of a cysteine at C6 could be followed by a nucleophilic substitution at C5, involving another thiol. The two sulfur atoms could then form a disulfide, which would regenerate the unmodified base (Supplementary Figure S19B, middle). In yet another scenario, the thiol reducing agent could attack the cysteine sulfur atom directly (Supplementary Figure S19B, bottom). Once a C base is formed by dehalogenation in the active site of the methyltransferase, subsequent methylation with SAM as the methyl donor can proceed rapidly through the canonical pathway (Figure 3B).

### Inefficient dehalogenation and methyltransferase dependent methylation of 5FC and 5ClC DNA

It is widely believed that for the 5FC containing DNA, the formation of the covalent methyltransferase-DNA complex is followed by methyl transfer, leading to a stable 5,5-fluoromethyl-6-thio-cytosine intermediate. We have collected all experimental structures representing the complexes of DNA methyltransferases with 5FC bearing oligoduplexes (Supplementary Table S3). The representative structures together with the corresponding composite omit electron density or electrostatic potential maps for the flipped out base are shown in Supplementary Figure S20A. To account for the different weight of the substituents in the crystallographic and cryo-EM maps, we also calculated theoretical maps according to the recently published protocol (59) for idealized 5-fluoro, 5-methyl and 5,5-fluoromethyl-6,6-dihydro-cytosine using the AVOGADRO and GAMESS software (60,61) (Supplementary Figure S20B). All of the experimental structures deviate to some degree from the idealized planar 5-methyl or 5-fluoro substituted base, but the presence of 5,5-fluoromethyl substitution in the structures is ambiguous. All of the complexes were obtained in the presence of thiol reducing agents, but also, all were chromatographically purified prior to crystallization or vitrification. From this analysis, we concluded that the crystallographic and cryo-EM structures correspond to snapshots of an inefficient dehalogenation reaction in agreement with the experimental results obtained for M.MpeI and either 5FC or 5ClC containing DNA oligoduplexes (Figure 3GH and Supplementary Figure S11).

### Catalytic versatility of DNA methyltransferases

In the presence of the methyl donor SAM, C5 cytosine DNA methyltransferases catalyze the physiologically relevant C5 cytosine methylation. In the somewhat artificial circumstances, they promote a wide variety of reactions, that all have in common the destabilization of the C5-substituent. Reversal of cytosine methylation (46) and hydroxymethylation (42) by mammalian DNA methyltransferases have already been discussed. In addition, mammalian methyltransferases are also capable to release the carboxyl substituent from 5-carboxylcytosine (5caC) as CO_2_ (converted to bicarbonate and a proton in aqueous solution) in the absence of SAM, but they are not efficient to release formic acid from 5-formylcytosine (5fC). In our assays in the presence of SAM, this leads to substantial methylation of 5caC, but not 5fC (Supplementary Figure S21) (62). The data in this manuscript show that the repertoire of reactions that are catalyzed by DNA methyltransferases is even broader and includes also dehalogenation. The efficiency of dehalogenation is greatest for 5IC, somewhat lower for 5BrC, and too low to be directly detected in case of 5ClC and 5FC.

### ‘In’ and ‘out’ conformations

C5-DNA methyltransferases undergo major conformational changes in the region of the active site cysteine during the catalytic cycle according to molecular dynamics simulations (63). Fluorescence changes in stopped flow experiments have been interpreted as a closing of the pocket for the flipped base, followed by methyl transfer, and reopening of the pocket at the end of the catalytic cycle (29). The ‘in’ and ‘out’ conformations seen in the crystals are likely to represent snapshots of this cycle. The conformations are not specific to M.MpeI, but are also observed for M.HhaI and M.HaeIII, the structurally closest relatives of M.MpeI among prokaryotic DNA methyltransferases. In the case of DNMT1 methyltransferase, the ‘in’ conformation is observed for productive complexes, whereas the ‘out’ conformation is observed for the autoinhibited, inhibited, and DNA-free forms (Supplementary Figure S22, Supplementary Table S4).

The ‘out’ conformation has an accessible active site and represents the state of the enzyme that is able to accept a flipped base into the substrate binding pocket. In the crystal structures, this ‘out’ conformation is also seen for the reaction products or their analogues, i.e. for cytosine analogues with bulky 5-substituents. In solution, a product base with a C5 methyl group (or its analogue) would be ejected. In the crystal, this is not possible because of the lattice that enforces the DNA conformation, and so the active site loop gives way instead. The ‘in’ conformation is only seen for analogues of substrate C that either have little steric bulk at the C5 position (such as 5FC), or are small enough to be repositioned in the active site (5mZ). The higher affinity of enzymes for substrates than for products suggests that the ‘in’ form of the methyltransferase active loop should facilitate the formation of covalent (5FC), mixed (5mZ) or non-covalent (dhaC, before enzymatic methylation) protein-DNA complexes. This conclusion is consistent with earlier work indicating that a reduction in active site crowding is conducive to the formation of tighter protein-DNA complexes (53–55).

## Supplementary Material

gkae568_Supplemental_File

## Data Availability

The structures of M.MpeI in complex with the analogue containing DNA fragments and the corresponding structure factors were deposited at Protein Data Bank with the following accession codes: 8C56 for 5mZ, 8C57 for dhaC converted to 5m-dhaC, 8C58 for 5OHC, and 8C59 for 5BrC converted to 5mC.
