# Correction to ‘Cytosine analogues as DNA methyltransferase substrates’

**DOI:** 10.1093/nar/gkae647

**Published:** 2024-07-17

This is a correction to: Marek Wojciechowski, Honorata Czapinska, Joanna Krwawicz, Dominik Rafalski, Matthias Bochtler, Cytosine analogues as DNA methyltransferase substrates, *Nucleic Acids Research*, 2024; gkae568, https://doi.org/10.1093/nar/gkae568

Figures 4 and 5 were accidentally swapped at proof stage. The published article has been updated.

This correction does not affect the results, discussion and conclusions presented in the article.

