# Supplementary material for: Cytosine analogues as DNA methyltransferase substrates
Source: Nucleic Acids Res. 2024 Jul 5;52(15):9267–81. doi: 10.1093/nar/gkae568 (PMC11347137; doi:10.1093/nar/gkae568)
Supplement: gkae568_Supplemental_File [file gkae568_supplemental_file.pdf]

Supplementary Material for:

Cytosine analogues as DNA methyltransferase substrates

Marek Wojciechowski[1,2,#], Honorata Czapinska[1,3,#], Joanna Krwawicz[1,3,4,#],
Dominik Rafalski[1,3,#], Matthias Bochtler[1,3,§]

[1]*International Institute of Molecular and Cell Biology, Trojdena 4, 02-109 Warsaw, Poland*

[2]*Plant Breeding and Acclimatization Institute - National Research Institute, 05-870 Radzikow, Poland*

[3]*Institute of Biochemistry and Biophysics PAS, Pawinskiego 5a, 02-106 Warsaw, Poland*

[4]*Department of Biochemistry, University of Oxford, Oxford, UK*

[#]Equal contribution

[§]Corresponding author

Tel: +48225970732

e-mail: mbochtler@iimcb.gov.pl

Supplementary Figures

Fig. S1

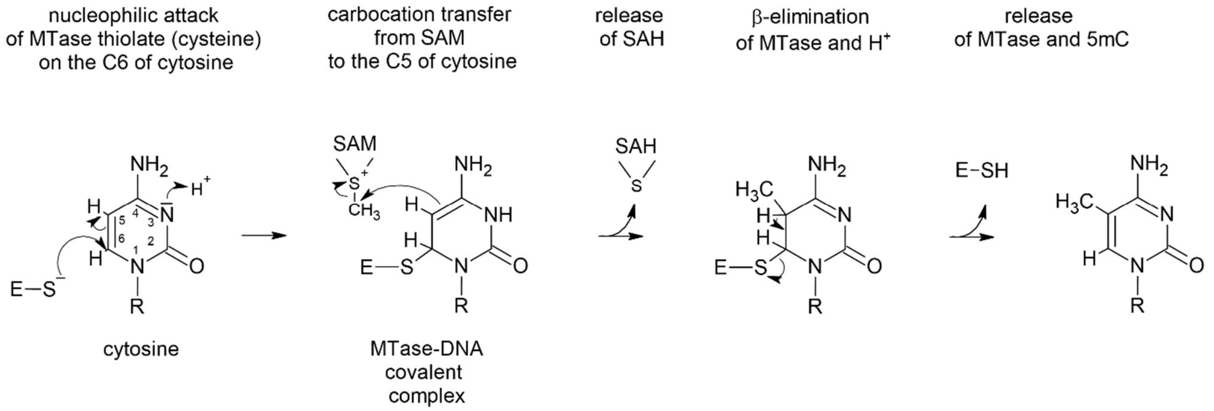

Fig. S1. Schematic representation of DNA methyltransferase catalyzed modification of cytosine to 5-methylcytosine.

Fig. S2

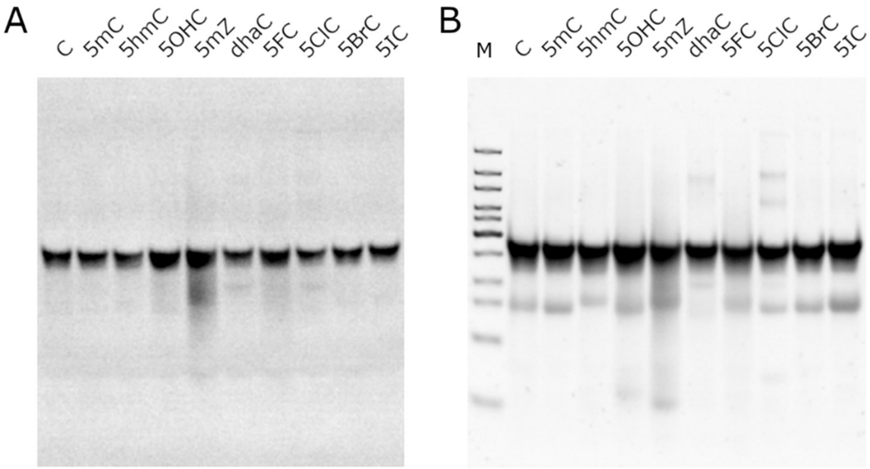

Fig. S2. Verification of the oligonucleotide quality. Oligonucleotide integrity was checked by 20% PAGE in TAE buffer and detected by **(A)** Cy3 fluorescence, then stained with **(B)** GelRed.

Fig. S3

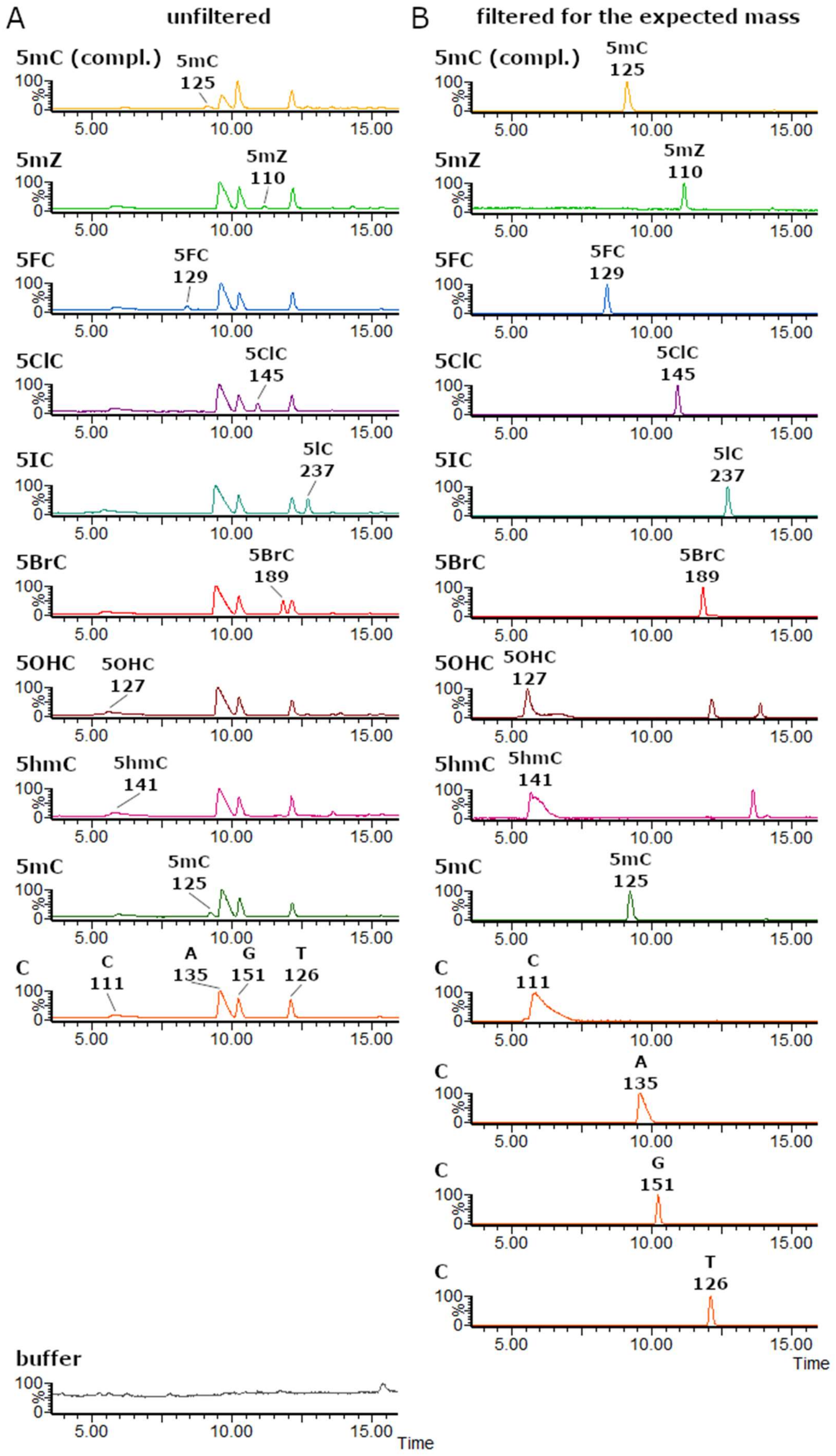
A
unfiltered
B
filtered for the expected mass
5mC (compl.)
5mC 125
5mZ
5mZ 110
5FC
5FC 129
5ClC
5ClC 145
5IC
5IC 237
5BrC
5BrC 189
5OHC
5OHC 127
5hmC
5hmC 141
5mC
5mC 125
C
C 111
A 135
G 151
T 126
buffer
100
%
0
5.00
10.00
15.00
Time

Fig. S3. MS of the total digest of modified 30-mer oligonucleotides. Oligonucleotides were degraded to 2′-deoxynucleoside monophosphates and dephosphorylated. The resulting mixtures were analyzed by LC-MS (MH+ mode). During ionization in the mass spectrometer, glycosidic bonds break, so that the free bases can be detected. Chromatograms are shown in the region where free bases elute: **(A)** without mass filtering, **(B)** with filtering for the expected mass of the protonated modified base. The identities of the expected modified bases are given next to the chromatograms. Bromine has two natural isotopes of nearly equal abundance that are separated by 2 Da in mass. For the filtering, the indicated mass of the 5BrC base was chosen. Oligoduplex sequences are indicated in Fig. 1 and Table S1. The chromatogram at the top shows the analysis for the 5mC containing oligo complementary to the one with the modification. All other chromatograms are for the strand carrying the modification. The masses of the unprotonated free bases are given next to the corresponding peaks.

Fig. S4

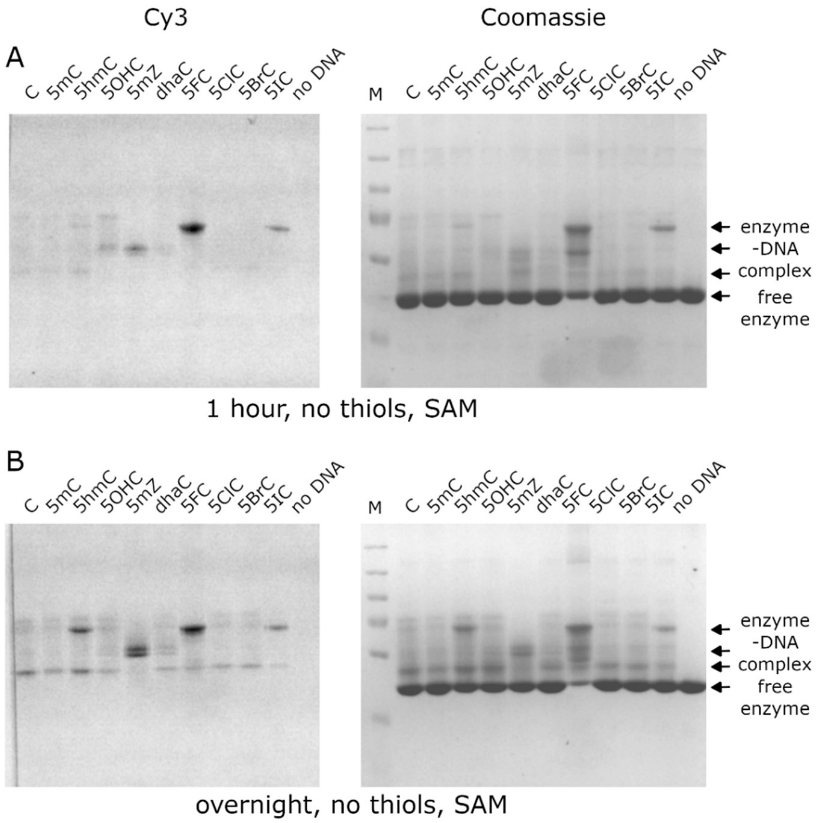

Fig. S4. Covalent complex formation between M.MpeI and modified oligoduplexes in the presence of S-adenosylmethionine (SAM) and absence of thiol reducing agents. The complex formation was monitored by 10% SDS PAGE after **(A)** 1 h and **(B)** overnight incubation. DNA was detected by Cy3 fluorescence (left), protein by Coomassie staining (right). Note that under electrophoresis conditions, DNA migrated out of the gel. ThermoFisher PAGERuler Prestained Protein Ladder was used as the protein size marker in all experiments (the expected protein size is 47.31 kDa).

Fig. S5

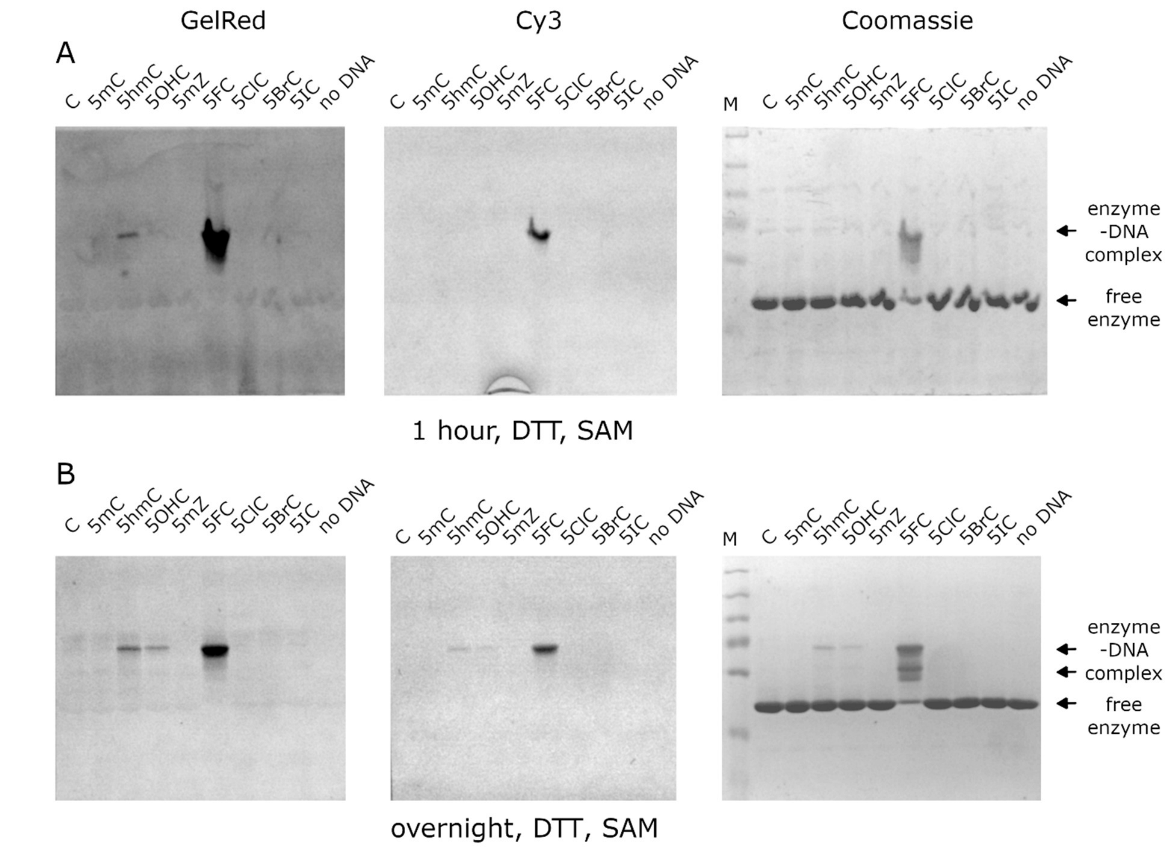

Fig. S5. Covalent complex formation between M.MpeI and modified oligoduplexes in the presence of SAM and a thiol reducing agent. Complex formation was monitored by 10% SDS PAGE after **(A)** 1 h and **(B)** overnight incubation. DNA was detected by GelRed staining (left) or Cy3 fluorescence (middle), protein was visualized using Coomassie staining (right).

Fig. S6

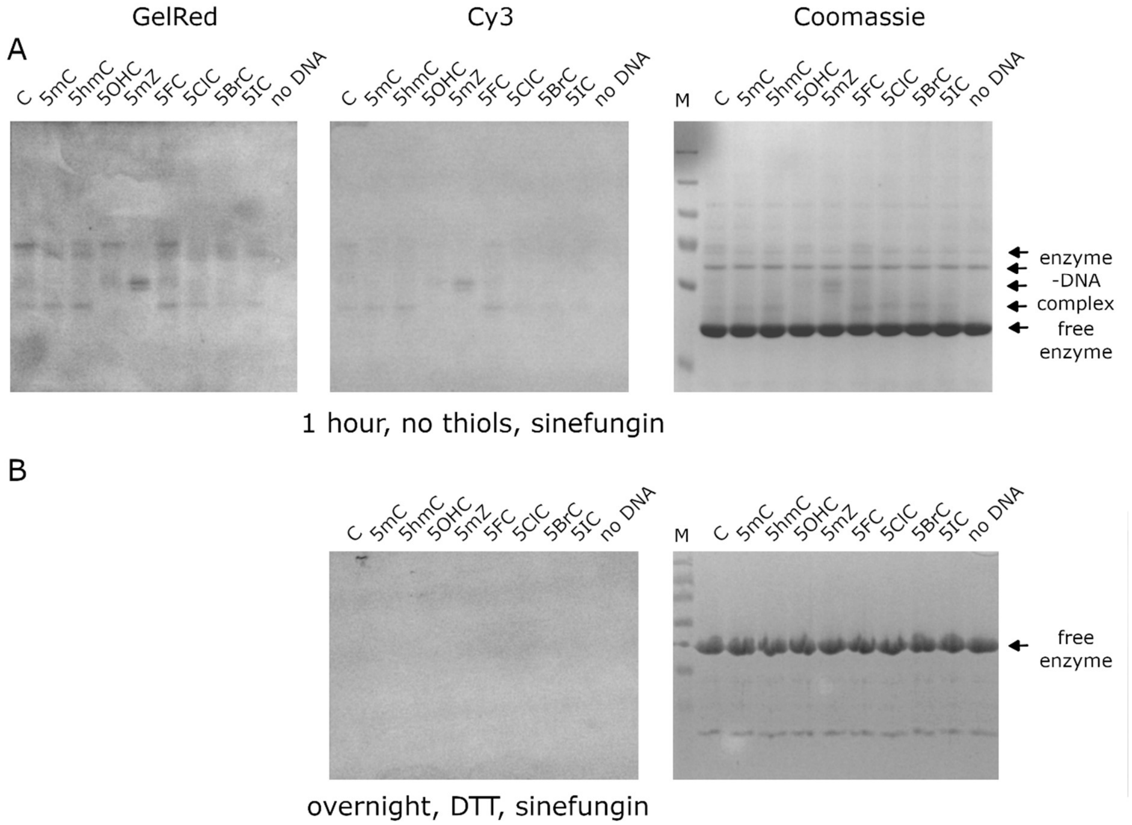

Fig. S6. Covalent complex formation between M.MpeI and modified oligoduplexes in the presence of sinefungin. The complex formation was detected by 10% SDS PAGE after **(A)** 1 h incubation in the absence of a thiol reducing agent **(B)** overnight incubation in the presence of DTT. DNA was detected with the help of GelRed (left) or Cy3 (middle), protein by Coomassie staining (right).

Fig. S7

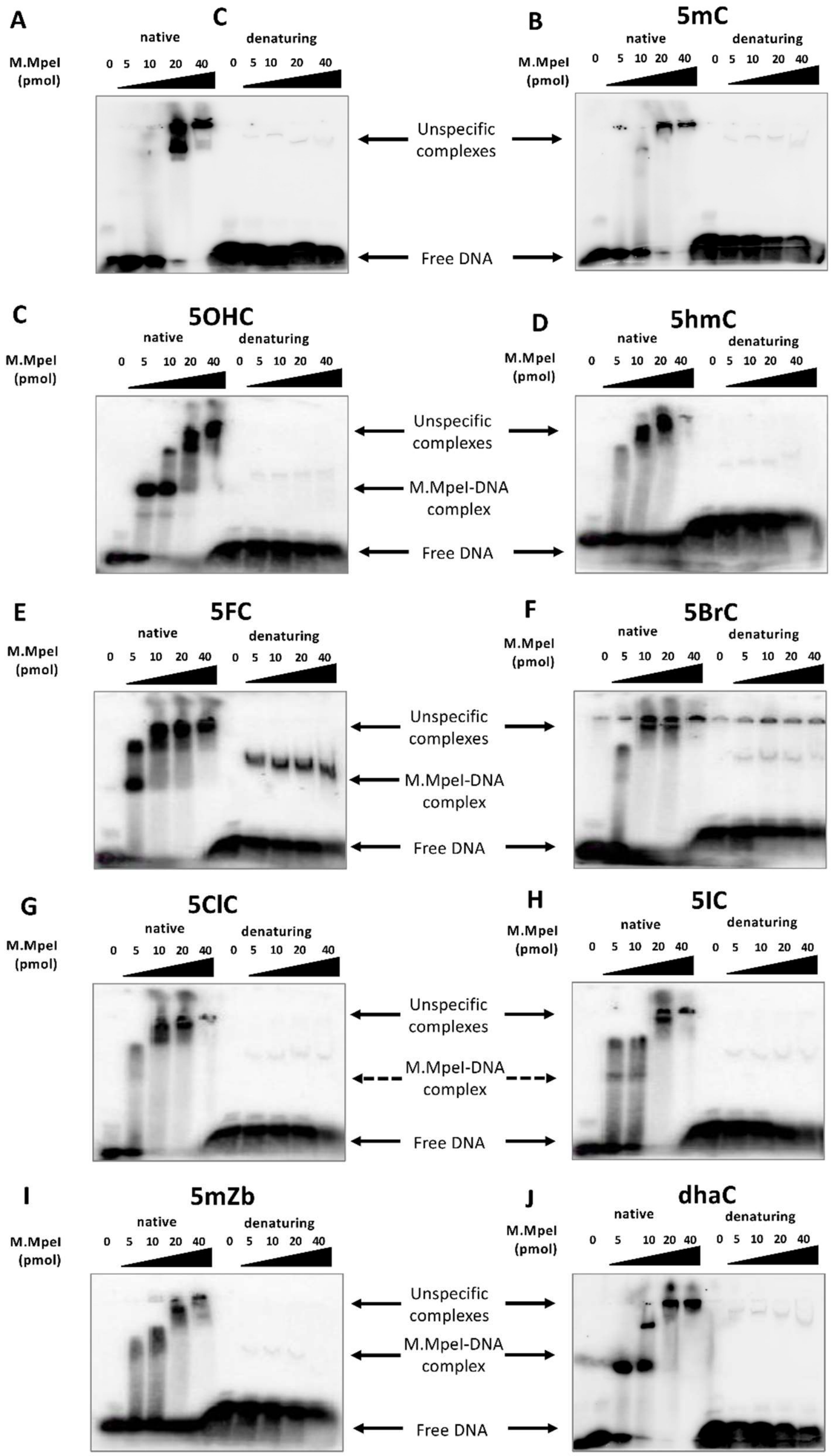

Fig. S7. Non-covalent versus covalent complex formation between M.MpeI and modified oligoduplexes. M.MpeI at increasing concentrations (0.5, 1, 2 and 4 µM) was incubated with modified oligoduplexes (1 µM) in the presence of SAM and DTT. Samples were loaded onto native 6% polyacrylamide TBE gels either directly after incubation (left, native conditions) or after additional treatment with 1% SDS at 65 °C for 5 min (right, denaturing conditions). ^{32}P labeled DNA was detected by autoradiography.

Fig. S8

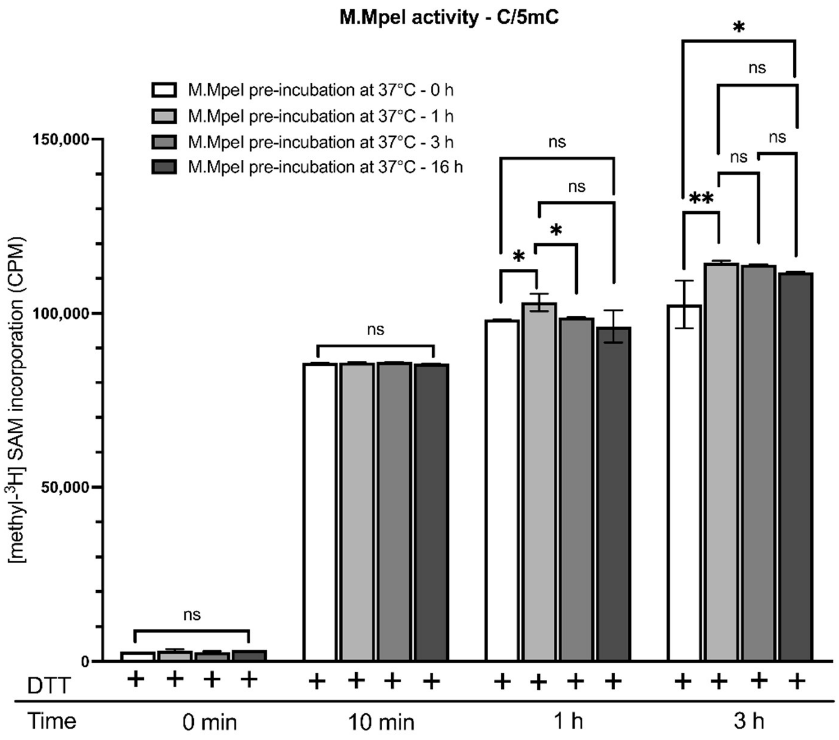

Fig. S8. Verification of M.MpeI integrity under experimental conditions. M.MpeI methyltransferase catalyzed transfer of the ^{3}H labelled CH_3 group from SAM to the hemimethylated target DNA was assayed before and after enzyme-DNA preincubation for 1, 3 and 16 h (radioactive SAM was added after the preincubation time). SAM and DNA were separated by Hybond filter binding or ethanol precipitation. The transfer of the radioactive CH_3 group was monitored with a scintillation counter. Multiple turnover conditions were used (100 pmol dsDNA and 20 pmol M.MpeI). The experiment was repeated three times, with two technical replicas each. Error bars corresponding to SD and the statistical significance by two-way ANOVA were calculated using Prism GraphPad software.

Fig. S9

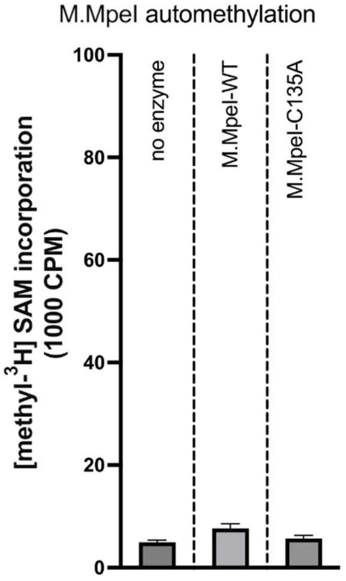

Fig. S9. Automethylation of WT M.MpeI and its inactive C135A variant in the absence of DNA. The reaction was run for 15 h. The assay was performed using anionic nitrocellulose membrane for protein binding instead of the cationic Hybond membrane used for nucleic acid binding.

Fig. S10

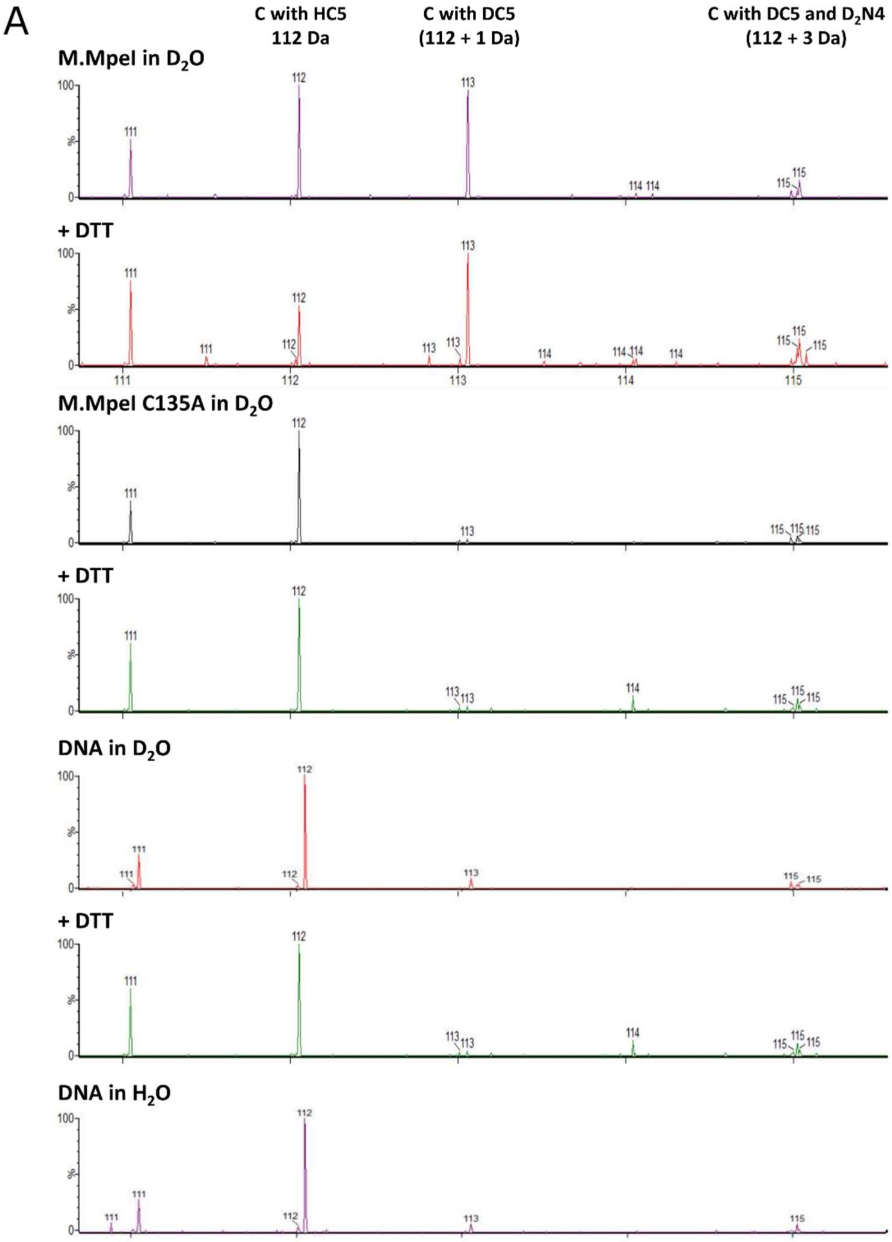

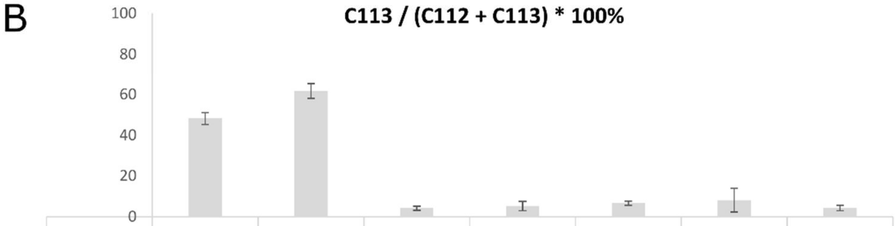

enzyme	M.MpeI	M.MpeI	M.MpeI C135A	M.MpeI C135A			
red agent		DTT		DTT		DTT	
oligo	C 5mC	C 5mC	C 5mC	C 5mC	C 5mC	C 5mC	C 5mC
solvent	D2O	D2O	D2O	D2O	D2O	D2O	H2O

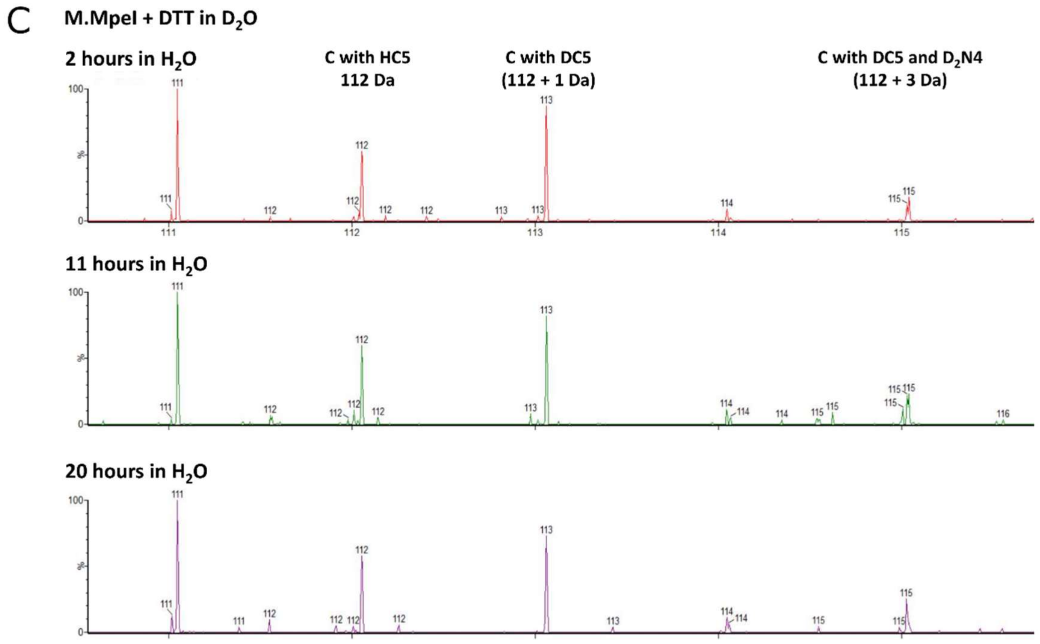

Fig. S10. M.MpeI catalyzed C5-hydrogen/deuterium exchange. Wild type M.MpeI or its C135A variant were incubated for 7 hours at 37 °C in D_2O. Reactions were stopped by a brief heating step. DNA was degraded, dephosphorylated to single nucleosides and analyzed by LC-MS at the peak of 2′-deoxycytidine elution from the column. **(A)** Mass spectra for the 111-115 Da mass range. Under the ionization conditions of mass spectrometry, the glycosidic bond of 2′-deoxycytidine breaks and therefore the mass for cytosine is recorded. The mass of 112 Da corresponds to the standard MH+ mass of cytosine before any exchange (C with HC5), the mass of 113 Da to the cytosine with C5 proton exchanged to deuterium (C with DC5), and of 115 Da to cytosine deuterated at C5 and N4 (C with DC5 and D_2N4). **(B)** Quantification of the experiment in (A). **(C)** Back-reaction control to estimate the maximum systematic error in the data due to the impossibility to analyze all samples at the exact same time. The data show that the back-reaction is very minor.

Fig. S11

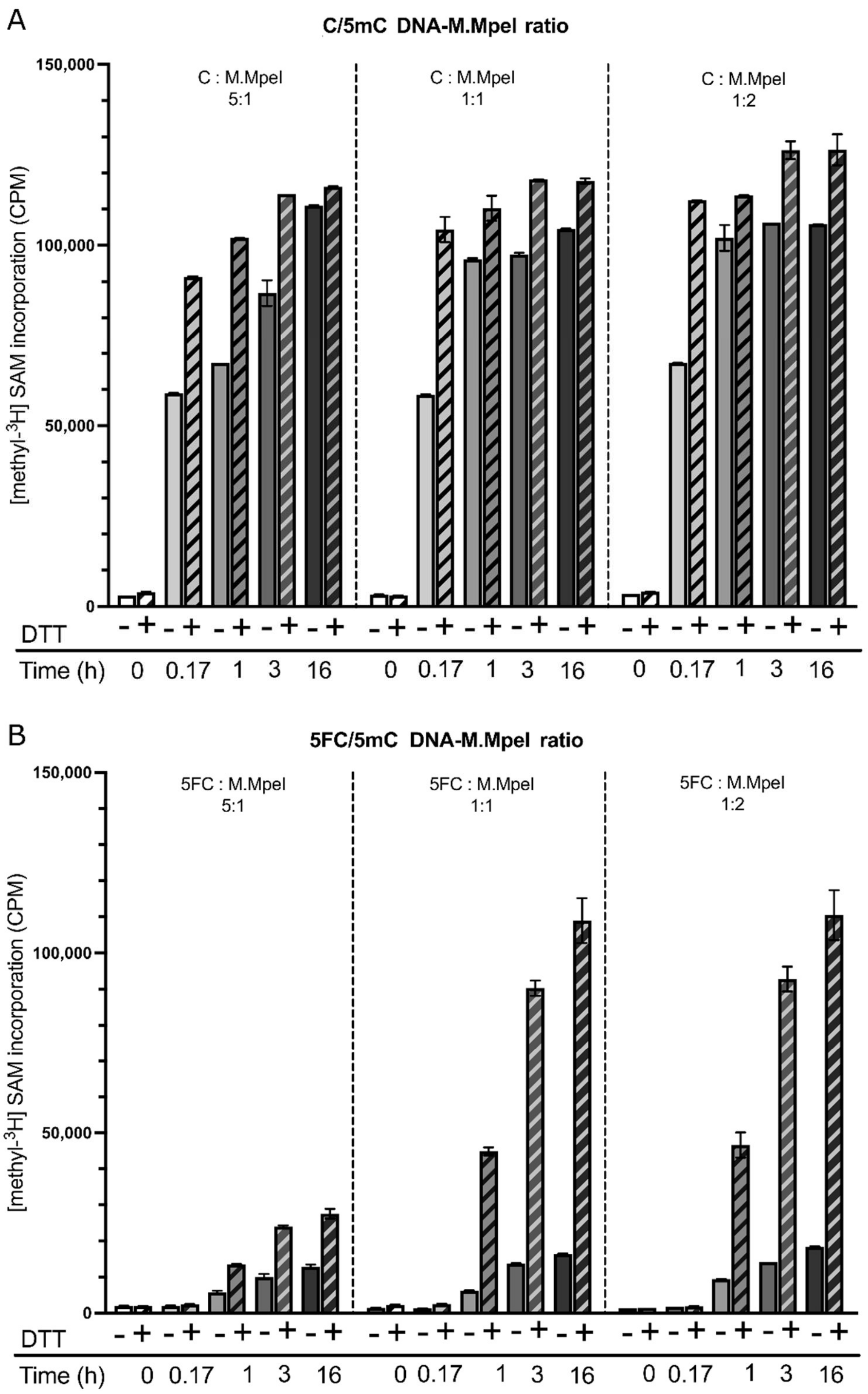

Fig. S11. Dependence of the methyl transfer on the M.MpeI - DNA stoichiometry. M.MpeI catalyzed the transfer of the ^{3}H labelled CH_3 group from SAM to the hemimethylated DNA containing either **(A)** C or **(B)** 5FC in the substrate strand. 100 pmol of DNA and 20, 100 or 200 pmol of M.MpeI were used. SAM and DNA were separated by filter binding or ethanol precipitation. The transfer of the radioactive CH_3 group was monitored using a scintillation counter. The experiment was repeated three times with two technical replicas each. Error bars corresponding to SD were calculated using Prism GraphPad Software.

Fig. S12

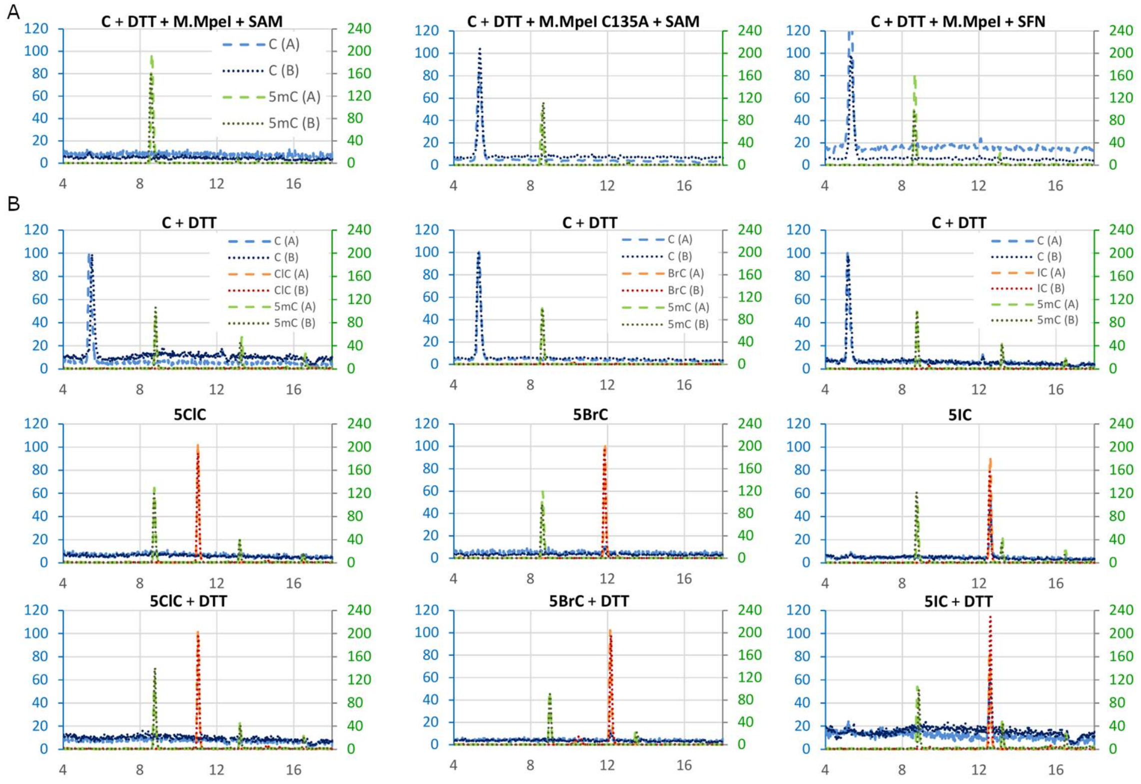
A
C + DTT + M.MpeI + SAM
C + DTT + M.MpeI C135A + SAM
C + DTT + M.MpeI + SFN
C (A)
C (B)
5mC (A)
5mC (B)
B
C + DTT
ClC (A)
ClC (B)
BrC (A)
BrC (B)
IC (A)
IC (B)
5ClC
5BrC
5IC
5ClC + DTT
5BrC + DTT
5IC + DTT

C

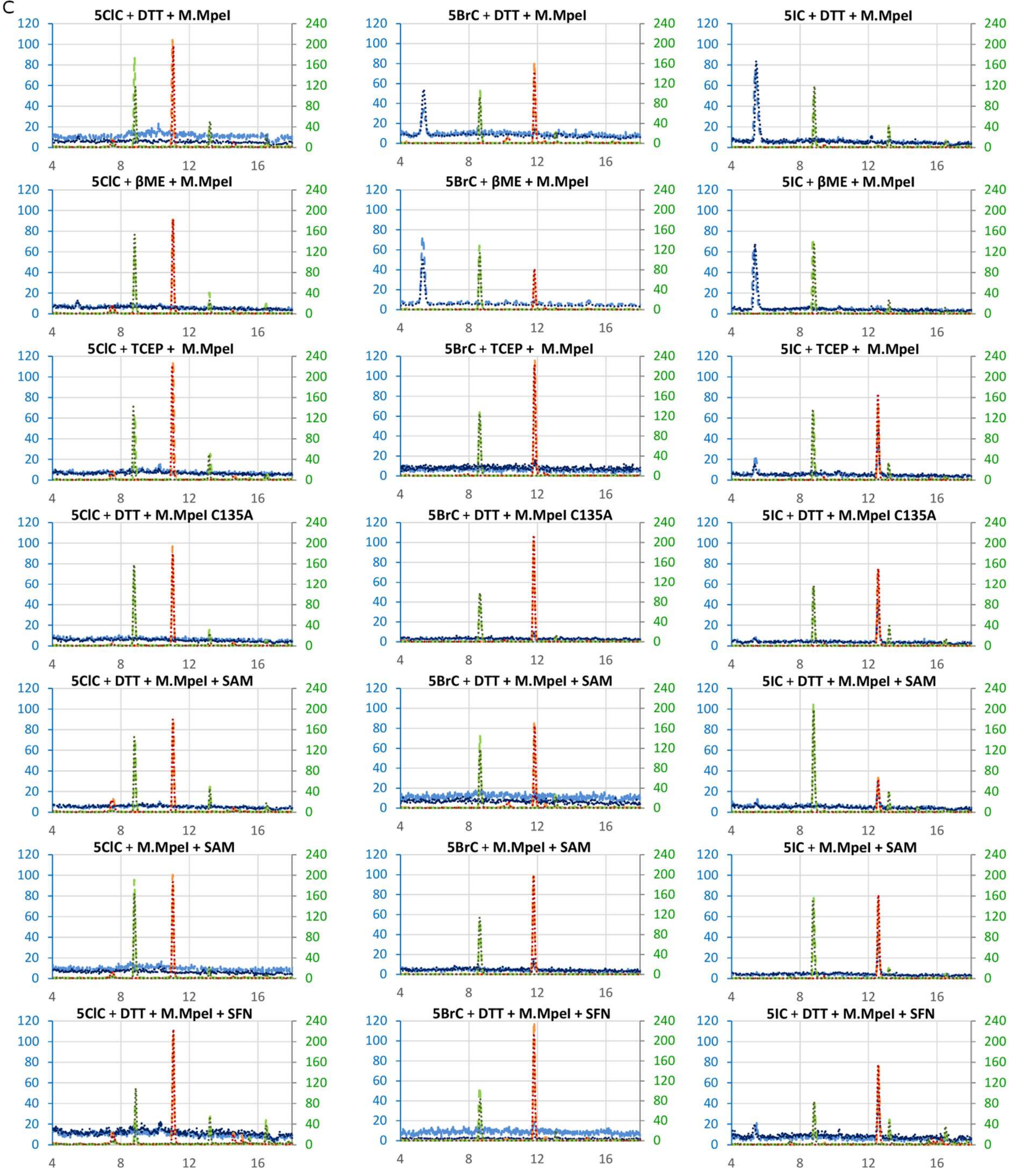

Fig. S12. M.MpeI and small molecule thiol nucleophile driven dehalogenation. DNA in the samples was degraded to single nucleotides, dephosphorylated and analyzed by LC-MS. The LC traces shown for C and 5mC are filtered for the expected masses. All reactions were scaled to the signal from a G base. The ordinate values were chosen so that the peak heights for the C or 5mC control in the DTT containing buffer are 100%. The halogen peaks were scaled with the peak height of the modified base in DTT containing buffer. The x-axes correspond to the elution time (min). The experiments were performed in duplicate and the average values were used for scaling. **(A)** Control experiments for SAM dependent M.MpeI activity show the expected disapearance of the C peak and doubling of the 5mC peak. As predicted the reaction is abolished by the catalytic mutation and the absence of SAM. **(B)** The controls used for normalization. **(C)** Experiments with the halogenated oligonucleotides.

Fig. S13

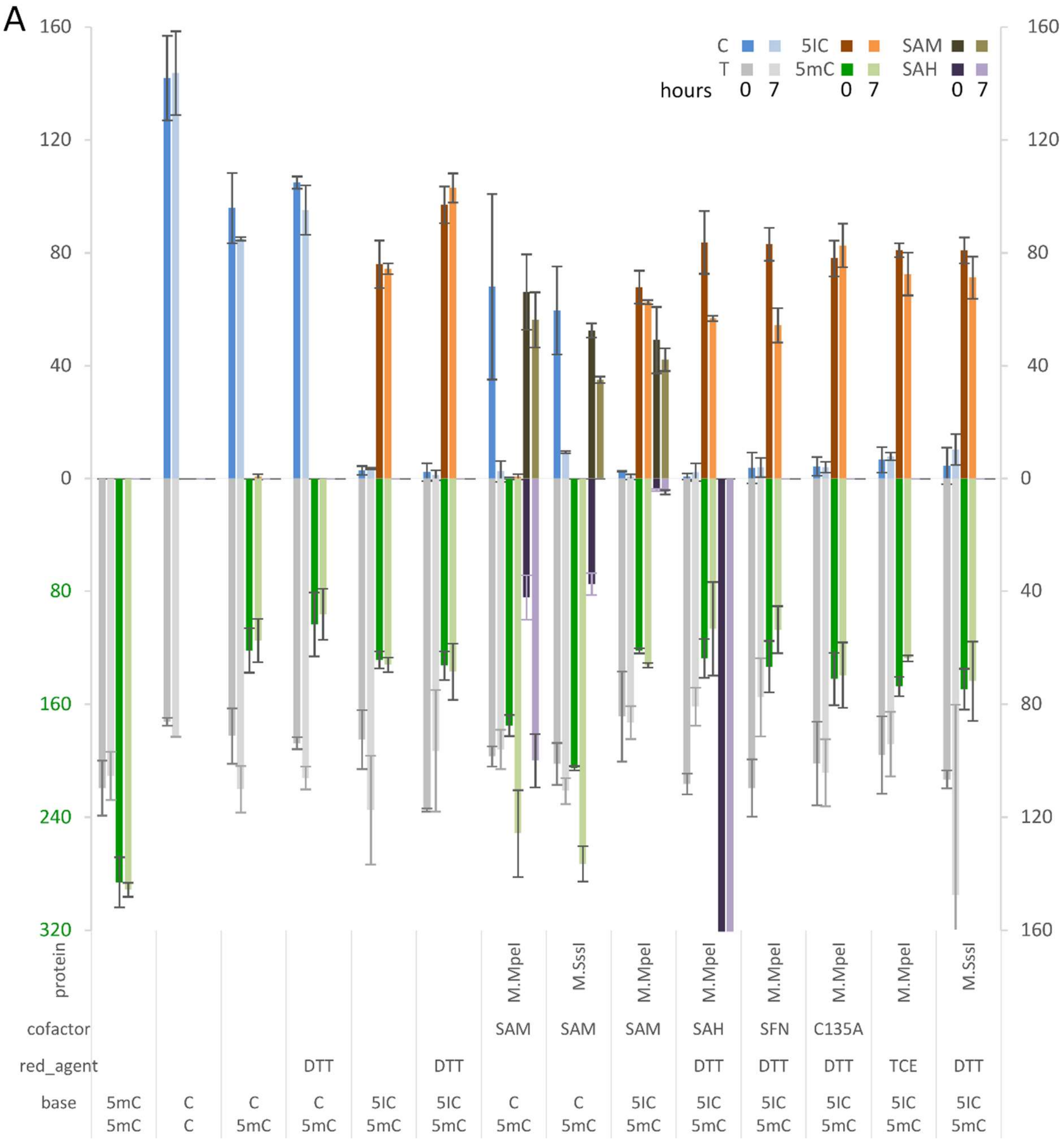

A
C
T
5IC
5mC
SAM
SAH
hours 0 7 0 7 0 7
160
120
80
40
0
80
160
240
320
40
80
120
160
protein
cofactor
red_agent
base
M.MpeI
M.SssI
SAM
SAH
SFN
C135A
DTT
TCE
5mC 5mC
C C
C 5mC
5IC 5mC

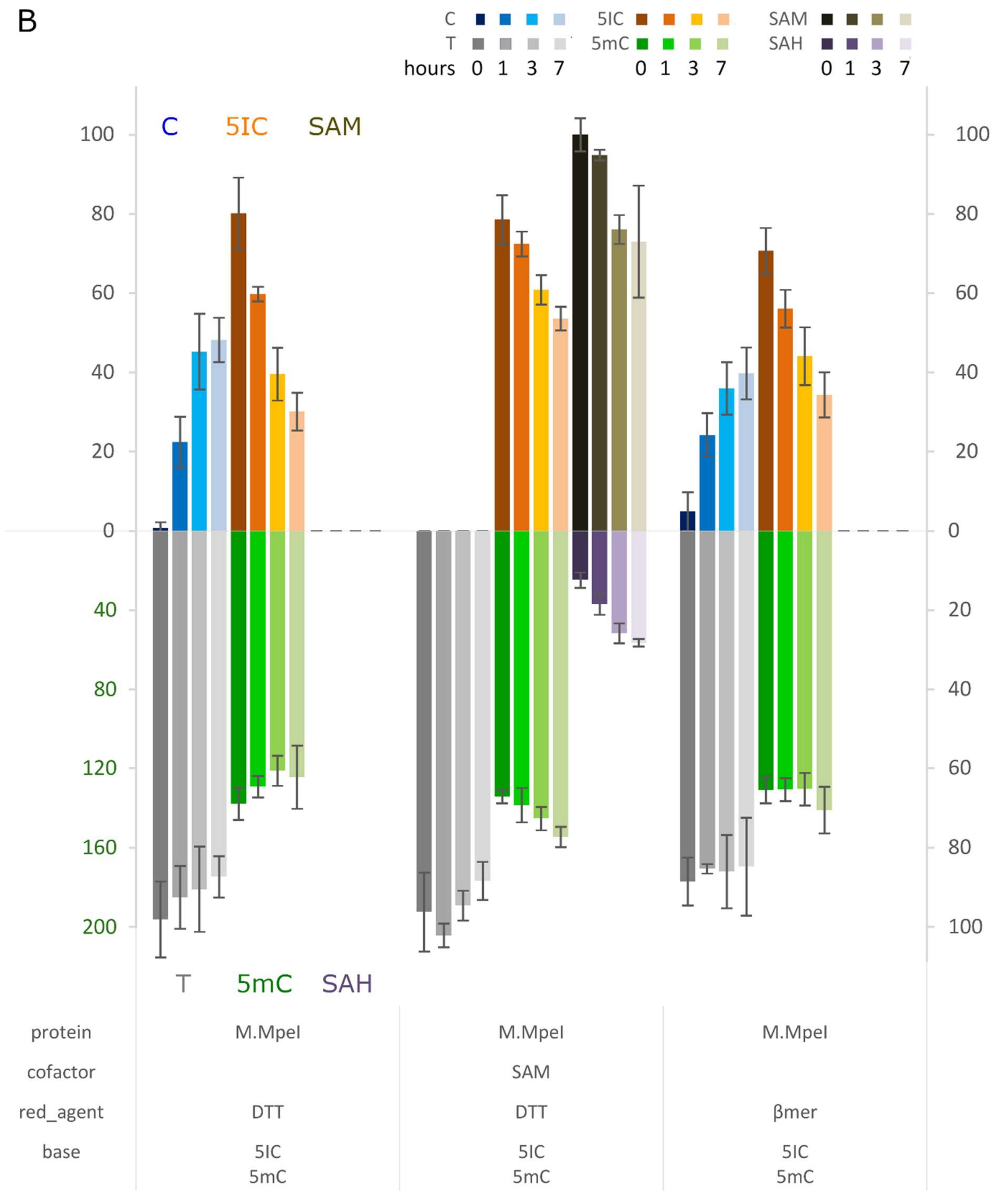

Fig. S13. Quantification of reaction substrates and products in the M.MpeI catalyzed dehalogenation (and methylation) reactions. (A) Endpoint assays with quantifications at 0 and 7 h. **(B)** Time courses (data collected at 0, 1, 3, 7 hours). After the indicated times, reactions were stopped by a brief heating step, and DNA was subjected to degradation to single nucleosides, dephosphorylation and LC-MS analysis. The chromatograms were filtered for the masses of the protonated forms of G, C, T, 5IC, 5mC, SAM, SAH (152, 112, 127, 238, 126, 298 and 385 Da). The sum of the peak areas for G was used for separate normalization of each spectrum. The areas for compounds were subsequently normalized to 100% for all spectra with the average values for C/5mC+DTT (C, T, 5mC), 5IC/5mC+DTT (5IC), M.MpeI+5IC/5mC+DTT+SAM at 0 h (SAM), M.MpeI+C/5mC+SAM at 7 h (SAH). Endpoint assays were performed in duplicate, time courses in triplicate. Results were averaged and standard errors were calculated. Note that the scale for 5mC differs from other series due to the presence of 5mC in the lower strand (lower left axes in green).

Fig. S14

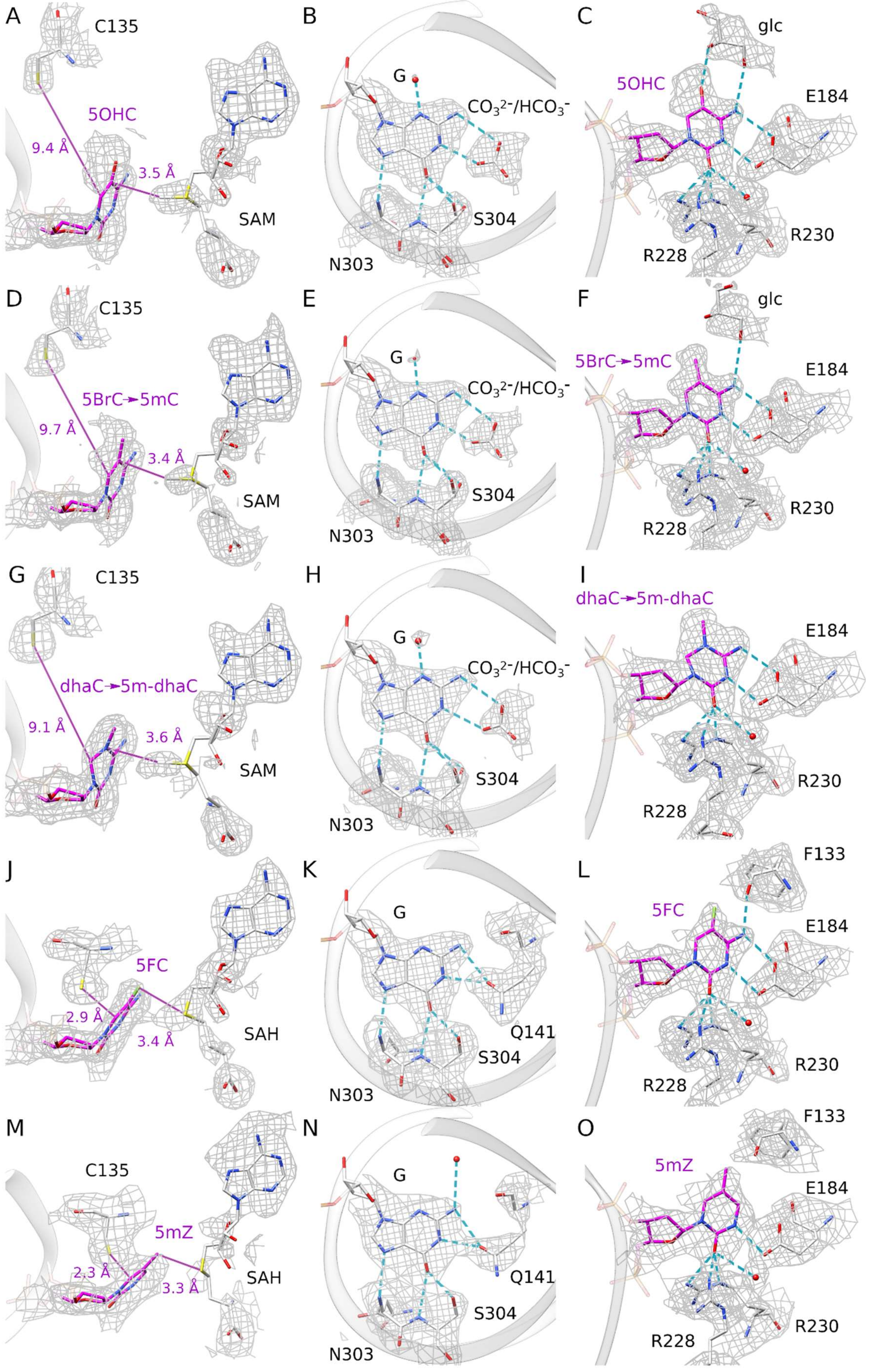

A
C135
5OHC
9.4 Å
3.5 Å
SAM
B
G
CO3 2-/HCO3 -
S304
N303
C
glc
5OHC
E184
R230
R228
D
C135
5BrC➛5mC
9.7 Å
3.4 Å
SAM
E
G
CO3 2-/HCO3 -
S304
N303
F
glc
5BrC➛5mC
E184
R230
R228
G
C135
dhaC➛5m-dhaC
9.1 Å
3.6 Å
SAM
H
G
CO3 2-/HCO3 -
S304
N303
I
dhaC➛5m-dhaC
E184
R230
R228
J
5FC
2.9 Å
3.4 Å
SAH
K
G
Q141
S304
N303
L
F133
5FC
E184
R230
R228
M
C135
5mZ
2.3 Å
3.3 Å
SAH
N
G
Q141
S304
N303
O
F133
5mZ
E184
R230
R228

Fig. S14. Conformation of the active site region of the M.MpeI complexes with cytosine analogues containing dsDNA. (**A-C**) 5-hydroxycytosine (5OHC), (**D-F**) 5-bromocytosine (5BrC) that got converted to 5mC prior or during the crystallization process, (**G-I**) 5,6-dihydro-5-azacytosine (dhaC) that got converted to 5m-dhaC, (**J-L**) 5-methylzebularine (5mZ), (**K-O**) 5-fluorocytosine (5FC)(1). The left column panels (**A,D,G,J,M**) depict the distances between the methyl donor co-substrate/co-product (SAM/SAH), the active site cysteine and the substrate/product base. For SAM the distances are to the C5/N5 of the base, for SAH to the C5 methyl group/fluorine atom, for Sγ of Cys135 to the C6 of the base. The middle column panels (**B,E,H,K,N**) show the guanine base that is estranged as a result of the substrate base flip. The right column panels (**C,F,I,L,O**) show the flipped substrate base. Glu184 makes two hydrogen bonds with the base as a result of the low pH of the crystallization buffer (pH 5.6). A glycerol molecule (glc) was tentatively modeled into the electron density because glycerol was used for cryo-protection and was abundant in the crystals. The carbonate (CO_3^{2-}) or bicarbonate (HCO_3^-) ion was probably derived from CO_2 captured from the air. As the pH of the crystallization drop (reservoir buffer pH 5.6) was close to the pK for the $HCO_3^-/CO_3^{2-}+H^+$ equilibrium (pKa=6.1) (2), a mixture was likely present. Composite omit maps were calculated using the CCP4 COMIT and FFT programs (3-5) and contoured at 1 rmsd.

Fig. S15

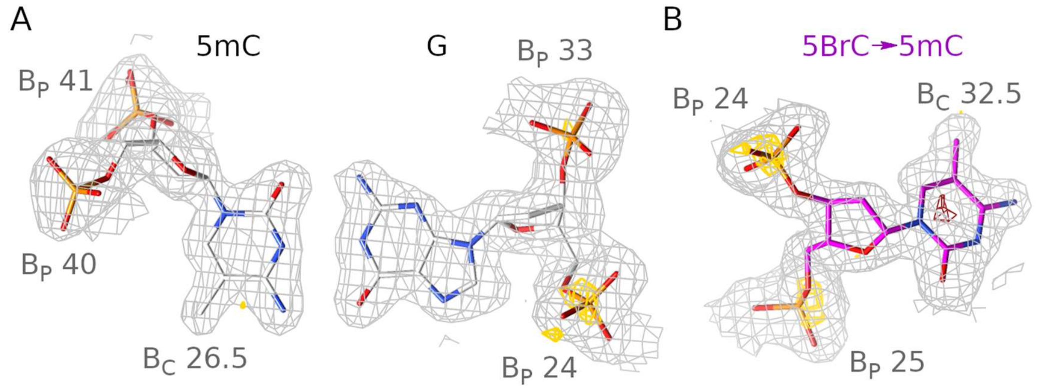

Fig. S15. Electron density maps for co-crystals of 5BrC containing DNA and M.MpeI. (**A**) 5mC:G pair with G in the substrate strand and 5mC in the complementary strand and (**B**) the flipped-out base bound in the substrate binding pocket. 5-bromocytosine bound as a substrate was converted to 5mC before or during the crystallization process as evidenced by the composite omit density contoured at 1 rmsd (grey), difference density at +/- 3 rmsd (green/red), and anomalous density at 3 rmsd (yellow). The B-factors of phosphorous atoms and carbons of the C5 methyl groups are indicated. Data collection was performed at the 0.9116 Å wavelength, where the theoretically predicted f'' values are approximately equal to 3.75 e for Br, 0.157 e for P and 0.003 e for C.

Fig. S16

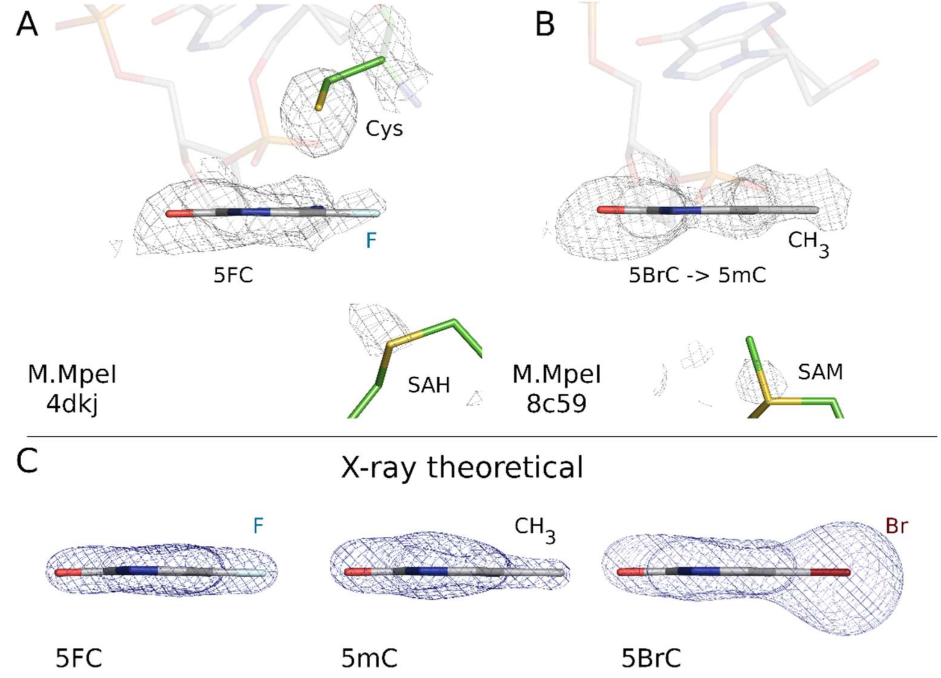

Fig. S16. Experimental electron densities for the complexes of M.MpeI with (A) 5FC and (B) 5BrC (converted to 5mC) containing oligoduplexes and (C) theoretically predicted maps. The electron densities (ED) correspond to the composite omit maps calculated after 10 REFMAC (6) refinement cycles for **(A)** previously published M.MpeI complex with 5FC containing DNA (1) and **(B)** the 5BrC->5mC containing complex presented in this work and contoured at 1.5 rmsd. **(C)** Theoretical densities were calculated as described (7) and contoured at either 5.5 (for 5FC and 5mC) or 3 rmsd (5BrC). The comparison of the observed and predicted densities suggests that the complexes correspond to pre- and post-reaction states. Note that due to the loop "out" conformation of M.MpeI in complex with 5BrC, the SAM most likely replaced SAH after the reaction.

Fig. S17

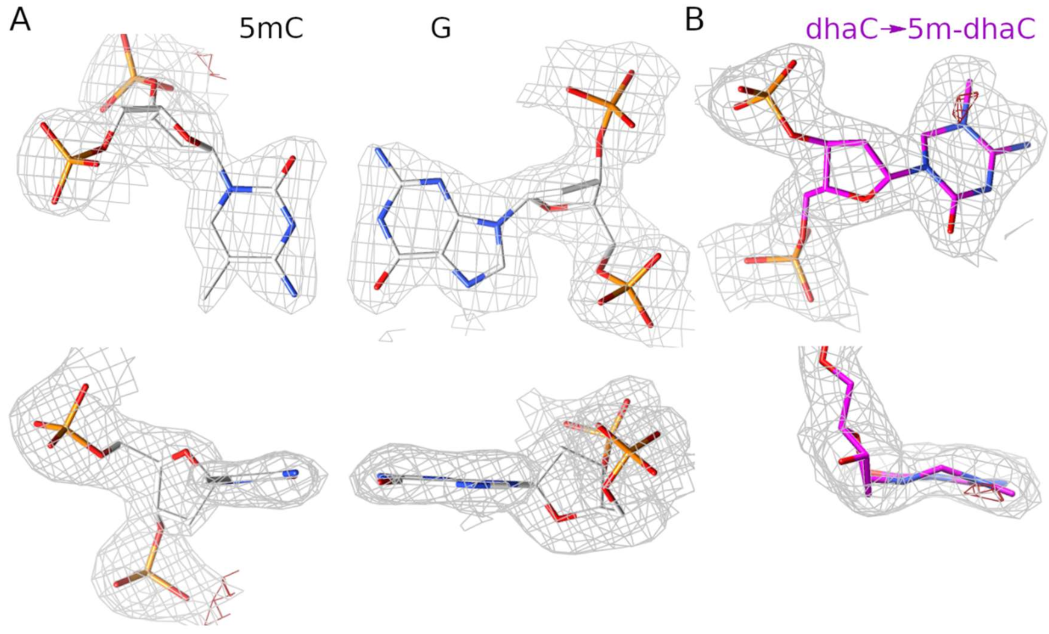

Fig. S17. Electron density maps for the co-crystal of dhaC containing DNA and M.MpeI. (A) 5mC:G pair with G in the substrate strand and 5mC in the complementary strand and **(B)** the flipped-out base in the substrate binding pocket. The originally added 5,6-dihydro-5-azacytosine was converted to 5m-dhaC prior to or during the crystallization process as indicated by the composite omit density contoured at 1 rmsd (grey), difference density contoured at +/- 3 rmsd (green/red), and biochemical experiments (Fig. 3). The top panels are in analogous orientation as in Fig. S15, the bottom panels are rotated ~90°.

Fig. S18

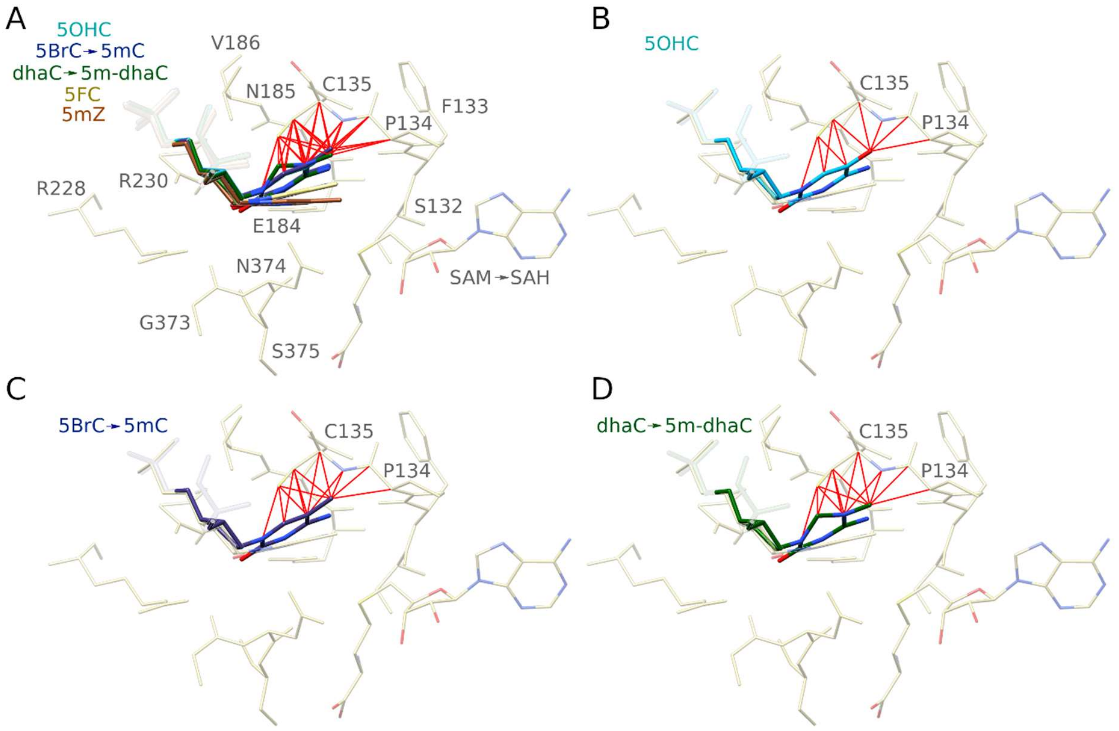

Fig. S18. Putative steric clashes between the base analogues in the M.MpeI substrate pocket and its active site loop. The catalytic site was modeled in the "in" conformation based on the 5FC-M.MpeI complex (1). **(A)** All studied complexes were superimposed to show the differences in base orientation relative to the active site. **(B-D)** Complexes with pronounced clashes are shown in separate panels. The residues of M.MpeI surrounding the flipped out analogues are shown as sticks (4.5 Å cutoff from the base was used). The red lines represent the contacts with more than 0.4 Å overlap. Such contacts were observed for **(B)** 5OHC, **(C)** 5mC (5BrC derived) and **(D)** 5m-dhaC (dhaC derived) for which the active site loop was present in the "out" conformation in the crystals. M.MpeI in complexes with 5FC and 5mZ DNA had the loop in the "in" conformation. In these cases steric conflicts did not occur, except with the active site Cys135 Sγ (not shown). The models were generated without adjustments of the protein and/or DNA, but some adaptive fit is expected in the actual complexes.

Fig. S19

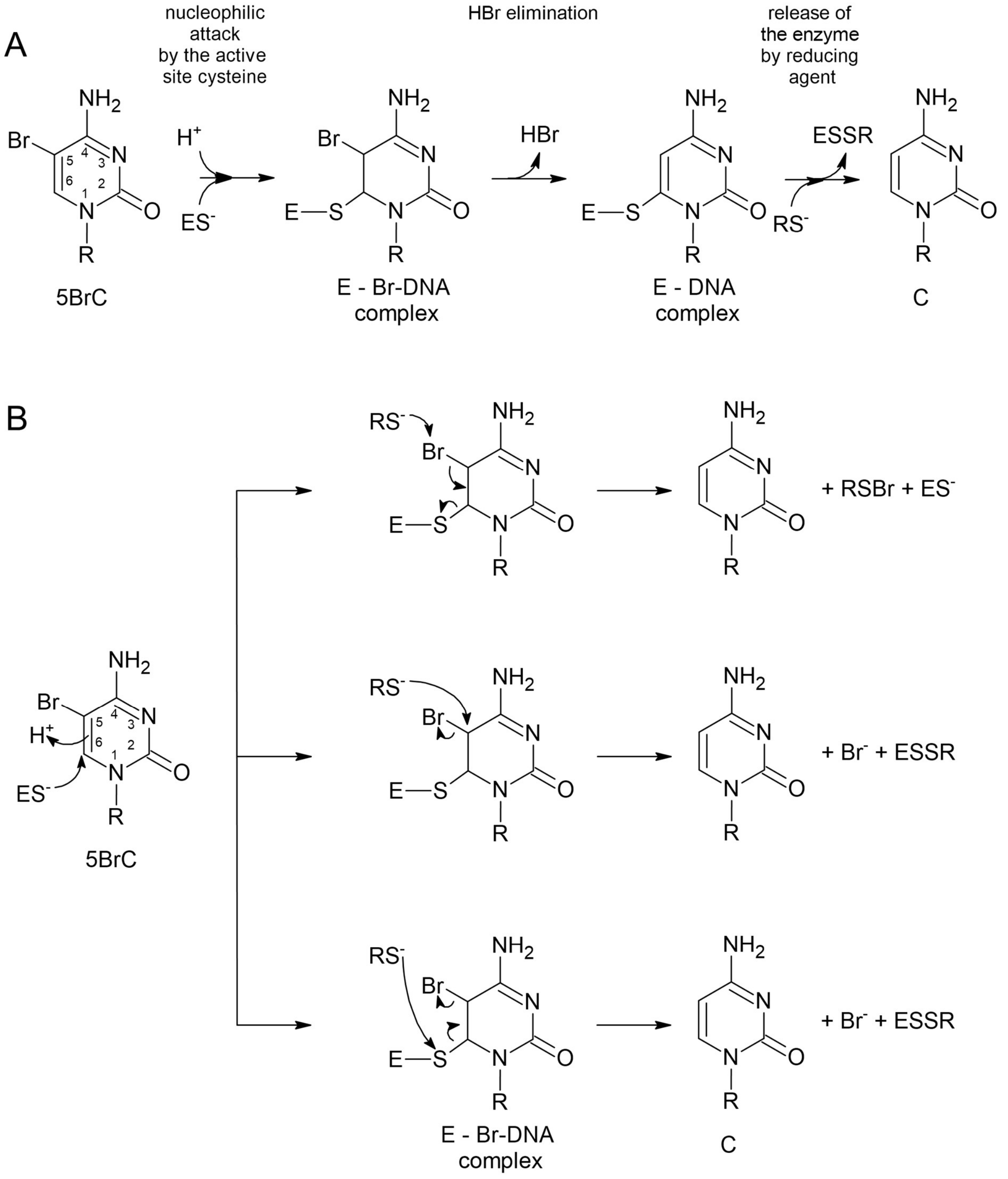

Fig. S19. Different scenarios for the dehalogenation of 5BrC (shown) or 5IC (analogous). (A) Possible mechanism involving formation of a covalent complex, followed by HBr elimination, and finally enzyme regeneration. **(B)** Alternative reaction mechanisms, based on literature about chemical dehalogenation of 5-halopyrimidines by small molecule thiol nucleophiles (8).

Fig. S20

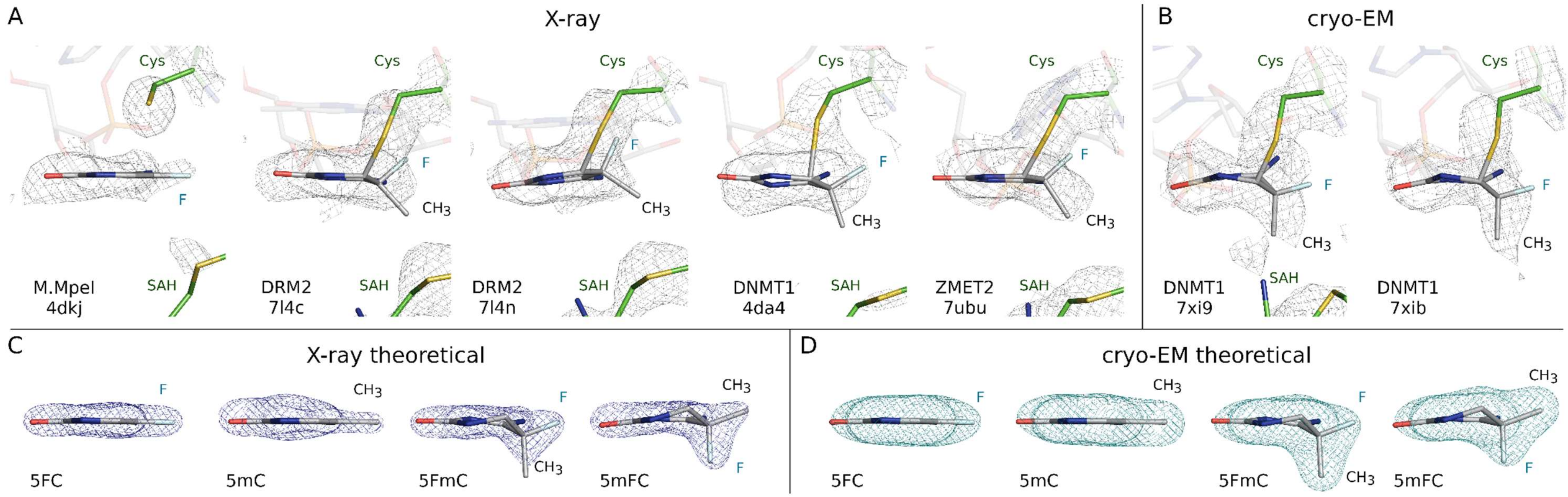

Fig. S20. Structurally characterized complexes of C5 DNA methyltransferases with 5FC containing oligoduplexes and the theoretic prediction of the expected electron density (ED) and electrostatic potential (EP) maps. (A) The representative crystal structures of MTase complexes with 5FC containing oligos. The atomic coordinates were downloaded from the PDB database (9). The complete list of structures is provided in Table S3. The ED maps correspond to the composite omit maps obtained with COMIT (3,4) after 10 cycles of restrained REFMAC refinement (6) and contoured at 1.5 rmsd. **(B)** The cryoEM reconstruction of the DNMT1 - 5FC oligoduplex complex (10). The two PDB depositions correspond to the two models obtained for different selections of images from the same data collection. The EP maps were contoured at 7.5 rmsd. **(C,D)** Theoretically predicted ED and EP maps were calculated as described (7) and contoured at either 5.5 (ED) or 4 (EP) rmsd.

Fig. S21.

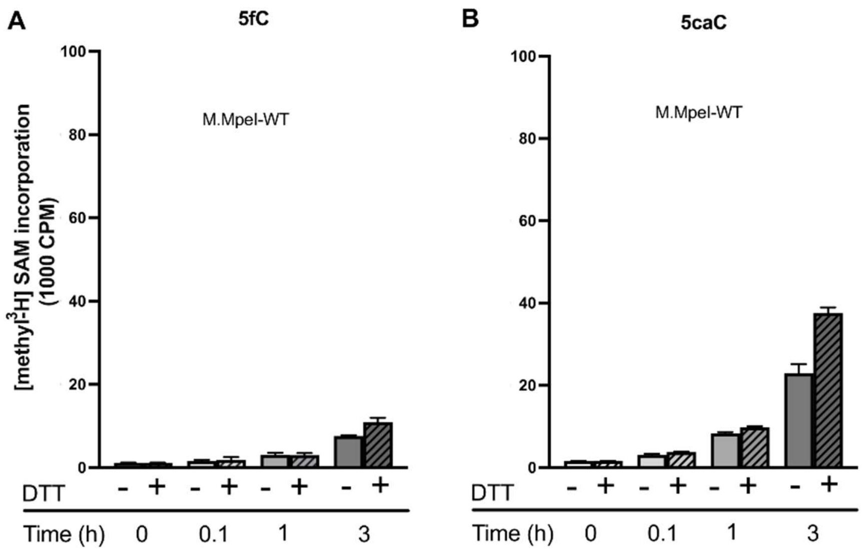

Fig. S21. Transfer of radiolabeled methyl groups to DNA containing (A) 5-formylcytosine (5fC) and (B) 5-carboxylcytosine (5caC). The assay was performed as in Fig. 3. In principle, the methyl transfer to 5caC could occur by formation of the methyl ester of the carboxylate. However, we consider this as unlikely for structural reasons. Instead, we interpret transfer of radiolabeled methyl to 5caC as methyltransferase catalyzed decarboxylation, followed by methylation. The higher reaction rate for 5caC than for 5fC likely reflects differences in the first step. 5fC is known to be largely refractory to methyltransferase driven release of formic acid, whereas 5caC is prone to methyltransferase catalyzed decarboxylation in the absence of SAM (11). In the presence of SAM, the newly formed C can be methylated. The slightly higher reaction rate in the presence of DTT than in its absence is due to a larger fraction of active M.MpeI with a reduced cysteine in the catalytic center.

Fig. S22

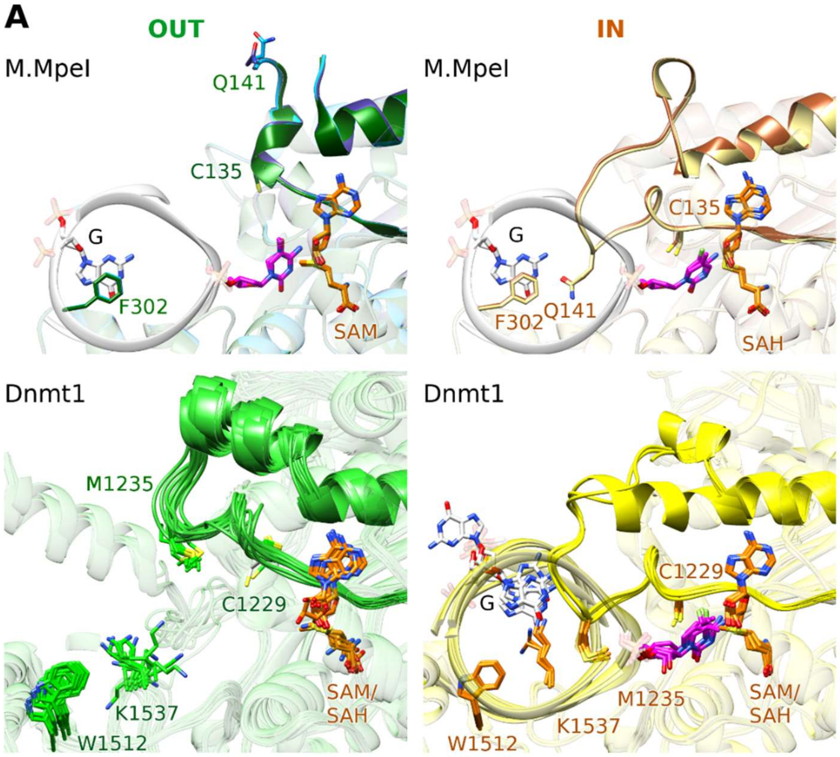

A
OUT
IN
M.MpeI
Q141
C135
G
F302
SAM
M.MpeI
C135
G
Q141
F302
SAH
Dnmt1
M1235
C1229
K1537
W1512
SAM/
SAH
Dnmt1
C1229
G
W1512
K1537
M1235
SAM/
SAH

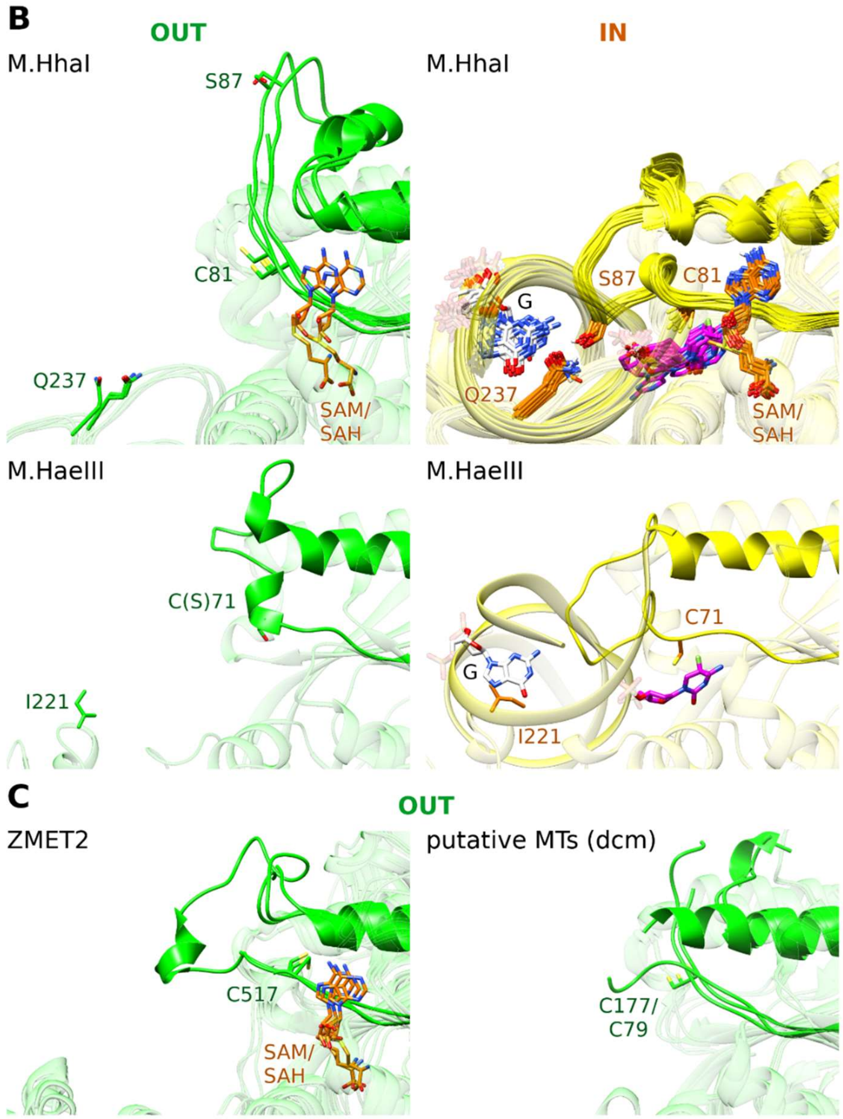

Fig. S22. Comparison of the "out" and "in" active site loop conformations in M.MpeI, DNMT1 and other methyltransferases (including putative ones). The set of methyltransferase structures is based on DALI scores (12) (Table S4). **(A)** In the case of M.MpeI (top row) the "out" conformation occurs in the presence of 5OHC, dhaC and 5BrC (converted to 5m-dhaC and 5mC, respectively) and the "in" conformation for 5FC and 5mZ. In the case of DNMT1 (second row) the "out" conformation occurs in the autoinhibited, inhibited, and DNA-free forms of the enzyme. The "in" conformation is observed for productive complexes. The state of the catalytic loop observed in the non-productive forms of mammalian DNMT1 methyltransferase is similar to the "out" form of the corresponding loop observed in M.MpeI analogue complexes. The state of the loop observed in the productive complex of DNMT1 resembles the catalytically competent "in" form of the loop in the M.MpeI structures. **(B)** A similar distribution of "in" and "out" conformations can also be observed for the bacterial methyltransferases M.HhaI and M.HaeIII. **(C)** For maize ZMET2 methyltransferase (13) and putative methyltransferases from *S. flexneri* (PDB IDs: 3LX6, 3ME5, unpubl.) and *E. coli* prophage (PDB ID: 7G7U, unpubl.) the productive complexes are missing and thus the "out" conformations can only be deduced based on structural similarities. The substrate/product base is indicated in magenta, SAM/SAH in orange. Non-productively bound DNA molecules are not shown.

Supplementary Tables

Table S1. List of phosphoramidites and oligonucleotides used in this study. (A) Phosphoramidite building blocks for base analogues, **(B)** DNA sequences and sources, **(C)** DNA applications.

(A)

**Compound name**	**Company**	**Catalog no.**
5-Me-dC-CE Phosphoramidite	Glen Research, VA United States	10-1060
5-I-dC-CE Phosphoramidite	Glen Research, VA United States	10-1081
5-Br-dC-CE Phosphoramidite	Glen Research, VA United States	10-1080
5-Me-2'-deoxyZebularine-CE Phosphoramidite	Glen Research, VA United States	10-1061
TMP-F-dU-CE Phosphoramidite*	Glen Research, VA United States	10-1016
5-Hydroxymethyl-dC-CE Phosphoramidite	Glen Research, VA United States	10-1062
5-OH-dC-CE Phosphoramidite	Glen Research, VA United States	10-1063
5-Chloro-dC-CE-Phosphoramidite	Biosearch Technologies, UK	BA0375

* postsynthetically converted to 5F-dC

(B)

**Name**	**Sequence (5' to 3')**	**Source(s)**
30-5mC-down	GGTTATTGTGTAAA**5mC**GCTGTGATAATGCTG	Purimex (Grebenstein, Germany)<br>FutureSynthesis (Poznan, Poland)
Cy3-30-5mC-down	**Cy3-**GGTTATTGTGTAAA**5mC**GCTGTGATAATGCTG	FutureSynthesis (Poznan, Poland)
30-C	CAGCATTATCACAG**C**GTTTACACAATAACC	Purimex (Grebenstein, Germany)<br>FutureSynthesis (Poznan, Poland)
30-5mC	CAGCATTATCACAG**5mC**GTTTACACAATAACC	Purimex (Grebenstein, Germany)<br>FutureSynthesis (Poznan, Poland)
30-5FC	CAGCATTATCACAG**5FC**GTTTACACAATAACC	Purimex (Grebenstein, Germany)<br>FutureSynthesis (Poznan, Poland)
30-5ClC	CAGCATTATCACAG**5ClC**GTTTACACAATAACC	FutureSynthesis (Poznan, Poland)
30-5BrC	CAGCATTATCACAG**5BrC**GTTTACACAATAACC	Purimex (Grebenstein, Germany)<br>FutureSynthesis (Poznan, Poland)
30-5IC	CAGCATTATCACAG**5IC**GTTTACACAATAACC	Purimex (Grebenstein, Germany)<br>FutureSynthesis (Poznan, Poland)
30-5OHC	CAGCATTATCACAG**5OHC**GTTTACACAATAACC	Purimex (Grebenstein, Germany)<br>FutureSynthesis (Poznan, Poland)
30-5hmC	CAGCATTATCACAG**5hmC**GTTTACACAATAACC	Purimex (Grebenstein, Germany)<br>FutureSynthesis (Poznan, Poland)
30-dhaC	CAGCATTATCACAG**dhaC**GTTTACACAATAACC	Purimex (Germany)
30-5mZ	CAGCATTATCACAG**5mZ**GTTTACACAATAACC	FutureSynthesis (Poznan, Poland)
14-5mC-down	TTCAG**5mC**GCATGTGG	Purimex (Germany)
14-C	CCACATG**C**GCTGAA	Purimex (Germany)
14-5BrC	CCACATG**5BrC**GCTGAA	Purimex (Germany)
14c-5mC-down	GTTCAG**5mC**GCATGTG	Purimex (Germany)
13-5FC	CACATG**5FC**GCTGAA	Purimex (Germany)
13-5BrC	CACATG**5BrC**GCTGAA	Purimex (Grebenstein, Germany)
13-5IC	CACATG**5IC**GCTGAA	Purimex (Grebenstein, Germany)

13-5OHC	CACATG**5OHC**GCTGAA	Purimex (Grebenstein, Germany)
13-5hmC	CACATG**5hmC**GCTGAA	Purimex (Grebenstein, Germany)
13-dhaC	CACATG**dhaC**GCTGAA	Purimex (Grebenstein, Germany)
13-5mZ	CACATG**5mZ**GCTGAA	Purimex (Germany)
14_5mC_R	AAATAT**5mC**GTTTATA	FutureSynthesis (Poznan, Poland)
14_C_F	TATAAA**C**GATATTT	FutureSynthesis (Poznan, Poland)
14_IC_F	TATAAA**5IC**GATATTT	FutureSynthesis (Poznan, Poland)
14_ClC_F	TATAAA**5ClC**GATATTT	FutureSynthesis (Poznan, Poland)
14_BrC_F	TATAAA**5BrC**GATATTT	FutureSynthesis (Poznan, Poland)

(C)

dsDNA substrate	Application	Concentration in reaction	Reaction volume
30-C / Cy3-30-5mC-down	SDS-PAGE complex formation analysis	3.75 µM	20 µl
30-5mC / Cy3-30-5mC-down			
30-5FC / Cy3-30-5mC-down			
30-5ClC / Cy3-30-5mC-down			
30-5BrC / Cy3-30-5mC-down			
30-5IC / Cy3-30-5mC-down			
30-5OHC / Cy3-30-5mC-down			
30-5hmC / Cy3-30-5mC-down			
30-dhaC / Cy3-30-5mC-down			
30-5mZ / Cy3-30-5mC-down			
30-C / ^{32}P-30-5mC-down	Electrophoretic mobility shift assay	1 µM	10 µl
30-5mC / ^{32}P-30-5mC-down			
30-5FC / ^{32}P-30-5mC-down			
30-5ClC / ^{32}P-30-5mC-down			
30-5BrC / ^{32}P-30-5mC-down			
30-5IC / ^{32}P-30-5mC-down			
30-5OHC / ^{32}P-30-5mC-down			
30-5hmC / ^{32}P-30-5mC-down			
30-dhaC / ^{32}P-30-5mC-down			
30-5mZ / ^{32}P-30-5mC-down			
30-C / 30-5mC-down	Methyl transfer	3.33 µM	30 µl
30-5mC / 30-5mC-down			
30-5FC / 30-5mC-down			
30-5ClC / 30-5mC-down			
30-5BrC / 30-5mC-down			
30-5IC / 30-5mC-down			
30-5OHC / 30-5mC-down			
30-5hmC / 30-5mC-down			
30-dhaC / 30-5mC-down			
30-5mZ / 30-5mC-down			
14-C / 14-5mC-down	RP-HPLC	10 µM	20 µl
14-5BrC / 14-5mC-down			
13-5FC / 14c-5mC-down	Crystallography	100 µM	-
13-5BrC / 14c-5mC-down			

13-5IC/14c-5mC-down			
13-5OHC / 14c-5mC-down			
13-5hmC / 14c-5mC-down			
13-dhaC / 14c-5mC-down			
13-5mZ / 14c-5mC-down			
14_5mC_R/14_C_F	Mass Spectrometry	16.67 µM	30 µl
14_5mC_R/14_IC_F			
14_5mC_R/14_ClC_F			
14_5mC_R/14_BrC_F			

Table S2. The dhaC tautomer ground state free energies. The energies were calculated in water in kcal/mol with GAMESS (14) using Roothaan-Hartree-Fock (RHF), hybrid RHF-DFT (Becke, 3-parameter, Lee–Yang–Parr, B3LYP) or Moller–Plesset (MP) method.

Calculation method / dhaC tautomer	RHF		B3LYP		MP
	6-31 G(d)	6-31 G(d,p)	6-31 G(d)	6-31 G(d,p)	6-31 G(d)
N3-H	-281649.21	-281661.47	-283167.31	-283177.66	-282477.27
N5-H	-281649.21	-281661.63	-283165.99	-283176.42	-282474.55

Table S3. Structural studies of C5 DNA methyltransferases in complex with 5FC containing oligoduplexes. The structures included in Fig. S20 are indicated in bold.

Protein	PDB ID	Exp. Meth	C mod	Planar	SAM SAH	DTT βME	SEC	pH	Experimental conditions	Res.	R_{free} [%]	R_{work} [%]	Source	SF/ map	DOI
DRM2	8T1U	XRAY	C49	Y	SAH	DTT - Reaction in 50 mM before SEC<br>5 mM in the crystallization drops	Y	7.0	0.2 M NH_4 citrate tribasic, 20% w/v PEG3350 pH 7.0	2.91	25.3	20.6	A. thaliana	Y	(15)
DRM2	7L4F	XRAY	C49	Y	SAH		Y	7	0.1 M Na acetate trihydrate pH 7.0, 12% w/v PEG3350	2.55	25.0	20.8	A. thaliana	Y	(16)
**DRM2**	7L4C	XRAY	C49	Y	SAH		Y	6.5	2% v/v Tacsimate pH 6.0, 0.1 M BIS-TRIS pH 6.5, 20% w/v PEG3350	2.11	21.5	18.5	A. thaliana	Y	(16)
DRM2	7L4H	XRAY	C49	Y	SAH		Y	7	0.2 M KI, 20% w/v PEG3350, pH 7.0	2.56	24.8	20.9	A. thaliana	Y	(16)
DRM2	7L4K	XRAY	C49	Y	SAH		Y	6.5	2% v/v Tacsimate pH 6.0, 0.1 M BIS-TRIS pH 6.5, 20% w/v PEG3350	2.61	24.0	21.3	A. thaliana	Y	(16)
**DRM2**	7L4N	XRAY	C49	n	SAH		Y	6.5	2% v/v Tacsimate pH 6.0, 0.1 M BIS-TRIS pH 6.5, 20% w/v PEG3350	2.25	21.0	18	A. thaliana	Y	(16)
DRM2	7L4M	XRAY	C49	y	SAH		Y	7	0.1 M Na formate pH 7.0, 12% w/v PEG3350	2.80	26.3	21.4	A. thaliana	Y	(16)
DNMT1	6W8V	XRAY	C49	?	SAH	DTT - Reaction in 10 mM before SEC<br>5 mM in the crystallization drops	Y	4.8	0.1 M Na citrate pH 4.8, 10 mM $ZnCl_2$	3.12	27.8	24.6	M. musculus	Y	(17)
DNMT1	6W8W	XRAY	C49	?	SAH		Y	4.8	0.1 M Na citrate, 10 mM $ZnCl_2$	3	28	23.8	M. musculus	Y	(17)
**DNMT1**	4DA4	XRAY	C49	n	SAH	DTT - Reaction in 4 mM before SEC<br>5 mM in the crystallization drops<br>3 mM TCEP form crystallization buffer	Y	4.5	3 mM TCEP, 0.1 M Na citrate pH 4.5, 277K	2.6	24.6	19.6	M. musculus	Y	(18)
**ZMET2**	7UBU	XRAY	C49	N	SAH	DTT - Reaction in 50 mM before SEC<br>5 mM in the crystallization drops	Y	5.5	0.1 M Na citrate pH 5.5, 15% w/v PEG6000	2.39	25.6	21.1	Z. mays	Y	(19)
M.HhaI	1MHT	XRAY	C36	nd	SAH - modelled, but crystallized with SAM	-	Y	7.25	BIS-TRIS-propane, NaCl, EDTA, pH 7.25, 289K	2.6		17.4	H. haemolyticus	N	(20)
M.HaeIII	1DCT	XRAY	C49	nd	-	DTT - Reaction in 1 mM DTT<br>crystallization buffer with 1 mM DTT	Y	6.5	100 mM MES pH 6.5, 120 mM CaCL, 9%-13% PEG3500,13% glycerol, 1 mM DTT, 277K	2.8	32.6	22.6	H. influenzae	N	(21)
**DNMT1**	7XI9	EM	EIX	N	SAH	DTT - Reaction 5 mM DTT	Y	7.5	20 mM Tris pH 7.5, 250 mM NaCl, 5 mM DTT				H. sapiens	Y	(10)
**DNMT1**	7XIB	EM	EIX	N	-								H. sapiens	Y	
**M.MpeI**	4DKJ	XRAY	C37	N	SAH - modelled, but crystallized with SAM	DTT - 1 mM DTT in the protein buffer	N	5.6	10% PEG 3350, 150 mM NaCl, 50 mM Na citrate pH 5.6, 25% v/v glycerol, pH 5.6, 294K	2.15	21.6	17.6	M. penetrans	Y	(1)

Table S4. Conformations of the active site loop in the C5-methyltransferase structures with the greatest similarity to M.MpeI.

(A) "in" conformation

Methyltransferase	Comment	Substrate/non-substrate base	Ligand	PDB-ID	Reference
DNMT1	productive complexes	5FC / 5mC	SAH	4DA4	(18)
		5FC / 5mC	SAH	6W8V	(17)
		5FC / 5mC	SAH	6W8W	(17)
		zebularine / 5mC	SAH	6X9I	(22)
		zebularine / 5mC	SAM	7SFG	(23)
M.HhaI		5FC / 5FC	SAH	1MHT	(20)
		C / C	SAH	3MHT	(24)
		5mC / 5mC	SAH	4MHT	
		C / 5mC	SAH	5MHT	(25)
		(5m)C (4'-thio) / 5mC	SAM	6MHT	(26)
		A / C	SAH	7MHT	(27)
		U (2′-deoxy) / C	SAH	8MHT	
		- / C	SAH	9MHT	
		5aC / 5mC	SAH	10MH	(28)
		C / C	SAH	2HR1	(29)
		C / C	SAH	2Z6Q	
		C / C	SAH	2I9K	(30)
		C / C	SAH	2Z6U	(31)
		C / C	SAH	2ZCJ	
		Z / C	SAH	1M0E	(32)
		C / C	SAH	2Z6A	(33)
		- / C	SAH	1SKM	(34)
		C / 5mC	SAH	2C7O	(35)
		C / 5mC	SAH	2C7P	
		C / 5mC	SAH	2C7Q	
		2-aminopurine / 5mC	SAH	2C7R	
		C / C	SAH	1FJX	(36)
		C / 5mC	SAH	2UYC	unpubl.
		C / 5mC	SAH	2UYH	
		C / 5mC	SAH	2UZ4	
		- / C	SAM	3EEO	
M.HaeIII		5FC / 5mC	-	1DCT	(21)

(B) "out" conformation

DNMT1	no DNA	-	SAH	3PT9	(37)
		-	SAH	5GUT	(38)
		-	-	5GUV	
	autoinhibited	-	-	3AV4	(39)
		-	SAH	3AV5	
		-	SAM	3AV6	
		-	SAH	3PTA	(37)
		-	SAH	3PT6	
		-	sinefungin	3SWR	unpubl.
		-	SAH	4WXX	(40)
		-	-	4YOC	(41)
		-	-	5WY1	(42)
	inhibited	Z / 5mC	-	6X9J	(22)
		Z / 5mC	-	6X9K	
		Z / 5mC	-	7SFC	(23)
		Z / 5mC	-	7SFE	
		Z / 5mC	-	7SFF	
M.HhaI	no DNA	-	SAM	1HMY	(43)
		-	SAH	1SVU	(44)
		-	SAM	2HMY	(45)
		-	-	5LOD	(46)
M.HaeIII		-	-	3UBT	(47)

References

1. Wojciechowski, M., Czapinska, H. and Bochtler, M. (2013) CpG underrepresentation and the bacterial CpG-specific DNA methyltransferase M.MpeI. *Proc Natl Acad Sci U S A*, **110**, 105-110.
2. Leader, D.P. (1979) A method of introducing the physiological carbon dioxide-bicarbonate buffer system to medical students. *Biochemical Education*, **7**, 37-38.
3. Vellieux, F.M.D. and Dijkstra, B.W. (1997) Computation of Bhat's OMIT maps with different coefficients. *J Appl Cryst*, **30**, 396-399.
4. Bhat, T.N. (1988) Calculation of an OMIT map. *J Appl Cryst*, **21**, 279-281.
5. Winn, M.D., Ballard, C.C., Cowtan, K.D., Dodson, E.J., Emsley, P., Evans, P.R., Keegan, R.M., Krissinel, E.B., Leslie, A.G., McCoy, A. *et al.* (2011) Overview of the CCP4 suite and current developments. *Acta Crystallogr D Biol Crystallogr*, **67**, 235-242.
6. Murshudov, G.N., Vagin, A.A. and Dodson, E.J. (1997) Refinement of Macromolecular Structures by the Maximum-Likelihood Method. *Acta Crystallographica Section D Biological Crystallography*, **53**, 240-255.
7. Bochtler, M. (2024) X-rays, electrons, and neutrons as probes of atomic matter. *Structure*, **32**, 630-643 e636.
8. Jacobson, D.G., Sedor, F.A., Sander, E.G. (1975) The dehalogenation of halocytosines by bisulfite buffers. *Bioorganic Chemistry*, **4**, 72-83.
9. Berman, H.M., Westbrook, J., Feng, Z., Gilliland, G., Bhat, T.N., Weissig, H., Shindyalov, I.N. and Bourne, P.E. (2000) The Protein Data Bank. *Nucleic Acids Research*, **28**, 235-242.
10. Kikuchi, A., Onoda, H., Yamaguchi, K., Kori, S., Matsuzawa, S., Chiba, Y., Tanimoto, S., Yoshimi, S., Sato, H., Yamagata, A. *et al.* (2022) Structural basis for activation of DNMT1. *Nat Commun*, **13**, 7130.
11. Liutkeviciute, Z., Kriukiene, E., Licyte, J., Rudyte, M., Urbanaviciute, G. and Klimasauskas, S. (2014) Direct decarboxylation of 5-carboxylcytosine by DNA C5-methyltransferases. *J Am Chem Soc*, **136**, 5884-5887.
12. Holm, L. (2022) Dali server: structural unification of protein families. *Nucleic Acids Res*, **50**, W210-W215.
13. Du, J., Zhong, X., Bernatavichute, Y.V., Stroud, H., Feng, S., Caro, E., Vashisht, A.A., Terragni, J., Chin, H.G., Tu, A. *et al.* (2012) Dual binding of chromomethylase domains to H3K9me2-containing nucleosomes directs DNA methylation in plants. *Cell*, **151**, 167-180.
14. Barca, G.M.J., Bertoni, C., Carrington, L., Datta, D., De Silva, N., Deustua, J.E., Fedorov, D.G., Gour, J.R., Gunina, A.O., Guidez, E. *et al.* (2020) Recent developments in the general atomic and molecular electronic structure system. *J Chem Phys*, **152**, 154102.
15. Chen, J., Lu, J., Liu, J., Fang, J., Zhong, X. and Song, J. (2023) DNA conformational dynamics in the context-dependent non-CG CHH methylation by plant methyltransferase DRM2. *J Biol Chem*, **299**, 105433.
16. Fang, J., Leichter, S.M., Jiang, J., Biswal, M., Lu, J., Zhang, Z.M., Ren, W., Zhai, J., Cui, Q., Zhong, X. *et al.* (2021) Substrate deformation regulates DRM2-mediated DNA methylation in plants. *Sci Adv*, **7**.
17. Adam, S., Anteneh, H., Hornisch, M., Wagner, V., Lu, J., Radde, N.E., Bashtrykov, P., Song, J. and Jeltsch, A. (2020) DNA sequence-dependent activity and base flipping mechanisms of DNMT1 regulate genome-wide DNA methylation. *Nat Commun*, **11**, 3723.
18. Song, J., Teplova, M., Ishibe-Murakami, S. and Patel, D.J. (2012) Structure-based mechanistic insights into DNMT1-mediated maintenance DNA methylation. *Science*, **335**, 709-712.
19. Fang, J., Jiang, J., Leichter, S.M., Liu, J., Biswal, M., Khudaverdyan, N., Zhong, X. and Song, J. (2022) Mechanistic basis for maintenance of CHG DNA methylation in plants. *Nat Commun*, **13**, 3877.
20. Klimasauskas, S., Kumar, S., Roberts, R.J. and Cheng, X. (1994) HhaI methyltransferase flips its target base out of the DNA helix. *Cell*, **76**, 357-369.
21. Reinisch, K.M., Chen, L., Verdine, G.L. and Lipscomb, W.N. (1995) The crystal structure of HaeIII methyltransferase convalently complexed to DNA: an extrahelical cytosine and rearranged base pairing. *Cell*, **82**, 143-153.
22. Pappalardi, M.B., Keenan, K., Cockerill, M., Kellner, W.A., Stowell, A., Sherk, C., Wong, K., Pathuri, S., Briand, J., Steidel, M. *et al.* (2021) Discovery of a first-in-class reversible DNMT1-selective inhibitor with improved tolerability and efficacy in acute myeloid leukemia. *Nat Cancer*, **2**, 1002-1017.

23. Horton, J.R., Pathuri, S., Wong, K., Ren, R., Rueda, L., Fosbenner, D.T., Heerding, D.A., McCabe, M.T., Pappalardi, M.B., Zhang, X. *et al.* (2022) Structural characterization of dicyanopyridine containing DNMT1-selective, non-nucleoside inhibitors. *Structure*, **30**, 793-802 e795.
24. O'Gara, M., Klimasauskas, S., Roberts, R.J. and Cheng, X. (1996) Enzymatic C5-cytosine methylation of DNA: mechanistic implications of new crystal structures for HhaL methyltransferase-DNA-AdoHcy complexes. *J Mol Biol*, **261**, 634-645.
25. O'Gara, M., Roberts, R.J. and Cheng, X. (1996) A structural basis for the preferential binding of hemimethylated DNA by HhaI DNA methyltransferase. *J Mol Biol*, **263**, 597-606.
26. Kumar, S., Horton, J.R., Jones, G.D., Walker, R.T., Roberts, R.J. and Cheng, X. (1997) DNA containing 4'-thio-2'-deoxycytidine inhibits methylation by HhaI methyltransferase. *Nucleic Acids Res*, **25**, 2773-2783.
27. O'Gara, M., Horton, J.R., Roberts, R.J. and Cheng, X. (1998) Structures of HhaI methyltransferase complexed with substrates containing mismatches at the target base. *Nat Struct Biol*, **5**, 872-877.
28. Sheikhnejad, G., Brank, A., Christman, J.K., Goddard, A., Alvarez, E., Ford, H., Jr., Marquez, V.E., Marasco, C.J., Sufrin, J.R., O'Gara, M. *et al.* (1999) Mechanism of inhibition of DNA (cytosine C5)-methyltransferases by oligodeoxyribonucleotides containing 5,6-dihydro-5-azacytosine. *J Mol Biol*, **285**, 2021-2034.
29. Shieh, F.K., Youngblood, B. and Reich, N.O. (2006) The role of Arg165 towards base flipping, base stabilization and catalysis in M.HhaI. *J Mol Biol*, **362**, 516-527.
30. Youngblood, B., Shieh, F.K., De Los Rios, S., Perona, J.J. and Reich, N.O. (2006) Engineered extrahelical base destabilization enhances sequence discrimination of DNA methyltransferase M.HhaI. *J Mol Biol*, **362**, 334-346.
31. Shieh, F.K. and Reich, N.O. (2007) AdoMet-dependent methyl-transfer: Glu119 is essential for DNA C5-cytosine methyltransferase M.HhaI. *J Mol Biol*, **373**, 1157-1168.
32. Zhou, L., Cheng, X., Connolly, B.A., Dickman, M.J., Hurd, P.J. and Hornby, D.P. (2002) Zebularine: a novel DNA methylation inhibitor that forms a covalent complex with DNA methyltransferases. *J Mol Biol*, **321**, 591-599.
33. Youngblood, B., Shieh, F.K., Buller, F., Bullock, T. and Reich, N.O. (2007) S-adenosyl-L-methionine-dependent methyl transfer: observable precatalytic intermediates during DNA cytosine methylation. *Biochemistry*, **46**, 8766-8775.
34. Horton, J.R., Ratner, G., Banavali, N.K., Huang, N., Choi, Y., Maier, M.A., Marquez, V.E., MacKerell, A.D., Jr. and Cheng, X. (2004) Caught in the act: visualization of an intermediate in the DNA base-flipping pathway induced by HhaI methyltransferase. *Nucleic Acids Res*, **32**, 3877-3886.
35. Neely, R.K., Daujotyte, D., Grazulis, S., Magennis, S.W., Dryden, D.T., Klimasauskas, S. and Jones, A.C. (2005) Time-resolved fluorescence of 2-aminopurine as a probe of base flipping in M.HhaI-DNA complexes. *Nucleic Acids Res*, **33**, 6953-6960.
36. Vilkaitis, G., Dong, A., Weinhold, E., Cheng, X. and Klimasauskas, S. (2000) Functional roles of the conserved threonine 250 in the target recognition domain of HhaI DNA methyltransferase. *J Biol Chem*, **275**, 38722-38730.
37. Song, J., Rechkoblit, O., Bestor, T.H. and Patel, D.J. (2011) Structure of DNMT1-DNA complex reveals a role for autoinhibition in maintenance DNA methylation. *Science*, **331**, 1036-1040.
38. Ye, F., Kong, X., Zhang, H., Liu, Y., Shao, Z., Jin, J., Cai, Y., Zhang, R., Li, L., Zhang, Y.W. *et al.* (2018) Biochemical Studies and Molecular Dynamic Simulations Reveal the Molecular Basis of Conformational Changes in DNA Methyltransferase-1. *ACS Chem Biol*, **13**, 772-781.
39. Takeshita, K., Suetake, I., Yamashita, E., Suga, M., Narita, H., Nakagawa, A. and Tajima, S. (2011) Structural insight into maintenance methylation by mouse DNA methyltransferase 1 (Dnmt1). *Proc Natl Acad Sci U S A*, **108**, 9055-9059.
40. Zhang, Z.M., Liu, S., Lin, K., Luo, Y., Perry, J.J., Wang, Y. and Song, J. (2015) Crystal Structure of Human DNA Methyltransferase 1. *J Mol Biol*, **427**, 2520-2531.
41. Cheng, J., Yang, H., Fang, J., Ma, L., Gong, R., Wang, P., Li, Z. and Xu, Y. (2015) Molecular mechanism for USP7-mediated DNMT1 stabilization by acetylation. *Nat Commun*, **6**, 7023.
42. Kanada, K., Takeshita, K., Suetake, I., Tajima, S. and Nakagawa, A. (2017) Conserved threonine 1505 in the catalytic domain stabilizes mouse DNA methyltransferase 1. *J Biochem*, **162**, 271-278.

43. Cheng, X., Kumar, S., Posfai, J., Pflugrath, J.W. and Roberts, R.J. (1993) Crystal structure of the HhaI DNA methyltransferase complexed with S-adenosyl-L-methionine. *Cell*, **74**, 299-307.
44. Dong, A., Zhou, L., Zhang, X., Stickel, S., Roberts, R.J. and Cheng, X. (2004) Structure of the Q237W mutant of HhaI DNA methyltransferase: an insight into protein-protein interactions. *Biol Chem*, **385**, 373-379.
45. O'Gara, M., Zhang, X., Roberts, R.J. and Cheng, X. (1999) Structure of a binary complex of HhaI methyltransferase with S-adenosyl-L-methionine formed in the presence of a short non-specific DNA oligonucleotide. *J Mol Biol*, **287**, 201-209.
46. Rondelet, G., Fleury, L., Faux, C., Masson, V., Dubois, J., Arimondo, P.B., Willems, L. and Wouters, J. (2017) Inhibition studies of DNA methyltransferases by maleimide derivatives of RG108 as non-nucleoside inhibitors. *Future Med Chem*, **9**, 1465-1481.
47. Didovyk, A. and Verdine, G.L. (2012) Structural origins of DNA target selection and nucleobase extrusion by a DNA cytosine methyltransferase. *J Biol Chem*, **287**, 40099-40105.
